# Visual cues in MI-BCI induce biases in EEG classification models with deep learning but not with standard machine learning

**DOI:** 10.3389/fnrgo.2026.1804143

**Published:** 2026-09-28

**Authors:** David Trocellier, Simon Kojima, Bernard N'kaoua, Fabien Lotte

**Affiliations:** 1 Inria Center at the University of Bordeaux, Talence, France; 2 LaBRI, Talence, France; 3 BPH, Bordeaux Population Health, University of Bordeaux, Bordeaux, France

**Keywords:** attribution maps, brain computer interfaces, deep learning, experimental bias, motor imagery

## Abstract

**Objective:**

Deep learning classification methods are increasingly popular in the brain-computer interface (BCI) field due to their ability to learn complex, non-linear representations from EEG data. Unlike traditional machine learning approaches, which typically rely on hand-crafted features, deep learning models autonomously extract spatio-temporal features directly from the raw or minimally preprocessed signal. However, this flexibility may come at the cost of reduced interpretability and increased susceptibility to confounding factors. This study aims to investigate whether the inclusion of task-related visual cues in the input EEG window biases deep learning models by leading them to exploit irrelevant features, thereby compromising their neurophysiological validity.

**Approach:**

This study investigates how including EEG signals measured during visual cue presentation on classification accuracy and feature extraction in motor imagery (MI)-based BCIs. Three classification pipelines were evaluated: CSP+LDA, a traditional machine learning approach; DeepConvNet, a convolutional neural network for EEG classification; and REVE, a Transformer-based EEG foundation model. All methods are trained and tested on two large scale open-access EEG datasets using two epoching strategies, with or without including the visual cue onset, to assess their impact on model performance and neurophysiological relevance. Attribution maps (e.g., Saliency maps) and CSP patterns are used to interpret the features learned by each model.

**Main results:**

Including the visual cue onset significantly improved the classification accuracy of DeepConvNet and REVE. However, attribution analysis showed that the model mainly relied on early parietal activations, most likely cue-related visual evoked potentials, rather than MI-related sensorimotor rhythms (ERD/ERS), which were more prominent when the cue onset was excluded. In contrast, CSP+LDA showed minimal accuracy changes across conditions, with consistent motor cortex activation patterns.

**Significance:**

These findings demonstrate that higher classification performance does not necessarily imply neurophysiological validity. In particular, deep learning models may exploit confounding signals, such as visual cue processing, which undermines their interpretability and applicability in asynchronous or real-world BCIs, in which there is no such cue. We advocate for systematically applying explainability tools in BCI research to ensure classifiers rely on task-relevant brain activity. Additionally, we emphasize the importance of excluding visual cue periods from EEG segments containing reposenses evoked by the visual cue onset from the input to deep learning models.

## 1 Introduction

A brain-computer interface is “*a system that measures brain activity and converts it in (nearly) real-time into functionally useful outputs to replace, restore, enhance, supplement, and/or improve the natural outputs of the brain, thereby changing the ongoing interactions between the brain and its external or internal environments. It may additionally modify brain activity using targeted delivery of stimuli to create functionally useful inputs to the brain*” (Slutzky et al., 2025). It can be used, for example, to control a wheelchair (Rebsamen et al., 2007), video games (Graimann et al., 2009), or BCI-based therapy for motor rehabilitation after a stroke (Nojima et al., 2012).

One of the most widely studied BCI paradigms is the Motor Imagery (MI)-based BCI. In this paradigm, users control a system by performing MI tasks, such as imagining hand or foot movements, which are detected through EEG signals. Indeed, both actual and imagined movements activate overlapping neural networks (Pfurtscheller and Lopes da Silva, 1999), allowing MI-based BCIs to exploit the modulation of the sensorimotor rhythms (SMR) over the motor cortex. This modulation is characterized by event-related desynchronization (ERD) and event-related synchronization (ERS), primarily observed in the μ (7–13 Hz) and β (15–30 Hz) frequency bands (Pfurtscheller and Solis-Escalante, 2009).

MI-BCIs typically rely on machine learning algorithms [such as Linear Discriminant Analysis (LDA), Support Vector Machines (SVM), Riemannian classifiers, or deep learning models Lotte et al., 2018] to decode ERD/ERS patterns from EEG signals. These patterns are generally extracted from the frequency band encompassing both μ and β rhythms (8–30 Hz), and are translated into control commands for an external system.

Over the past decade, deep learning techniques have gained popularity in the field of MI-BCI (Altaheri et al., 2023), thanks to their outstanding performance in other areas of research such as natural language processing, image recognition, and protein folding Jumper et al. (2021). These methods use architectures such as Convolutional Neural Networks (CNNs) and Recurrent Neural Networks (RNNs), which can learn complex, non-linear spatio-temporal features from raw or minimally preprocessed EEG data. Among the most widely used models for EEG classification are ShallowConvNet and DeepConvNet, both introduced by Schirrmeister et al. (2017), as well as EEGNet, developed by Lawhern et al. (2018). Each of these architectures is tailored to capture the distinct characteristics of EEG signals.

More recently, Transformer, an architecture based on the attention mechanism, has become increasingly popular across various fields (Vaswani et al., 2017). Transformer-based models have achieved high performance in domains such as computer vision, natural language processing, and speech recognition (Khan et al., 2022). In EEG decoding, various Transformer-based architectures have also been proposed in recent years (Abibullaev et al., 2023). Moreover, many EEG foundation models adopt Transformer-based architectures (Kuruppu et al., 2026). Among them, REVE has been reported to outperform conventional deep learning models and other EEG foundation models across multiple EEG datasets (El Ouahidi et al., 2025).

Another very widely adopted technique for real-time EEG classification (Lotte et al., 2018), is the combination of Common Spatial Patterns (CSP) and Linear Discriminant Analysis (LDA). This popularity comes from its computational efficiency and ease of calibration in online BCI settings (Ramoser et al., 2000). The CSP+LDA pipeline consists of two main stages: First a spatial filtering step that projects EEG signals onto a subspace where class-discriminative information is maximized, and second a classification step, typically performed with LDA, that assigns the power of the filtered signals to a class label.

In their review, Arpaia et al. (2022) compared the performance of classification algorithms from 2017 to 2021, analyzing both linear and non-linear methods. In this article, the authors categorize models into brain-inspired and non-brain-inspired approaches. Brain-inspired methods rely on architectures that mimic biological neural processes, such as artificial neural networks, deep neural networks, and spiking neural networks, to classify EEG signals. In contrast, non-brain-inspired methods are based on models that learn from data using hand-crafted features, typically involving algorithms such as SVM, decision trees, and LDA. Among the top 25% of classifiers on open source datasets, 39% were brain-inspired models for binary classification tasks, while this proportion increased to 78% in multi-class scenarios. Furthermore, the study reported a notable shift in methodological preferences over time. In 2017, approximately 30% of published approaches relied on non-brain-inspired methods. By 2021, this proportion increased significantly, with brain-inspired models comprising around 65% of all methods employed. These results show that brain-inspired methods, including deep learning models, are gaining popularity and often achieve higher classification accuracy compared to traditional approaches.

Despite this growing popularity (Arpaia et al., 2022), deep learning has not yet been conclusively established as the superior approach. Eder et al. (2024) found no significant difference between the performance of deep learning models and that of another state-of-the-art classifier, Riemannian geometry, in within-subject, cross-session, and cross-subject paradigms. The authors conclude that both approaches can be used similarly, and the chosen decoding approaches can be based on factors such as ease of implementation, computational efficiency, or individual preferences. Additionally, Chevallier et al. (2024) compared various machine learning models using MOABB, a framework aggregating multiple EEG datasets, and showed that Riemannian geometry-based methods outperform deep learning models in subject-specific training scenarios. They also note that deep learning models require a large number of training trials to achieve competitive performance. Interestingly, Tibrewal et al. (2022) shows that using deep learning models improves performance for inefficient BCI users, with low classification accuracy with linear models. This puts in perspectives that both approaches may have similar accuracy on average but work differently for a given user.

Even though Deep learning is not (yet) considered as the best classification method, it is becoming more and more popular. This recent popularity makes it interesting to evaluate in more detail the performance of these classifiers and their interactions with known confounding factors and bias in linear approaches. Indeed, due to the non-linear transformations applied by deep learning algorithms, and the differences in their feature extraction processes, the features learned by these models may differ from those derived using linear approaches. It is also known that, similarly to machine learning models, deep learning models may tend to extract features based on bias (Shah and Sureja, 2025) including data bias, algorithmic bias, and societal bias (Hendricks et al., 2018; Ryu et al., 2018). Bias in machine learning experiments can be defined as any systematic deviation introduced during data preprocessing, model design, training, or evaluation that results in inflated performance metrics, overfitting to non-task-related features, misinterpretation of the learned representations, or learning false representation of the world. Such bias may arise, for instance, from improper cross-validation strategies, data leakage (Riascos et al., 2024; Carlson et al., 2025), imbalanced dataset (Thölke et al., 2023), underepresentation of a part of the population (Hendricks et al., 2018), or neglecting the neurophysiological plausibility of the features extracted by the model. The extraction of implausible features may be impacted by confounding factors in the experimental setup, such as auditory distractors (Horowitz et al., 2021), visual processing load (Brouwer et al., 2015) or physiological artifacts like eye movements, muscle activity or heart rate (Brouwer et al., 2015; Fatourechi et al., 2007).

A previous study by Jacinto et al. (2009) demonstrated that it is possible to classify visual processing EEG features between left and right visual cues with high accuracy. This classification was achieved using an epoching window ranging from 200 ms before to 400 ms after the onset of the visual cue. To accomplish this, the authors employed a linear model optimized to discriminate between distinct event-related potential (ERP) components while preserving the temporal structure of the signal, which is critical for accurate ERP analysis (Schiff et al., 2005). Furthermore, they observed that presenting the visual cue on only one half of the screen, either left or right, resulted in higher classification accuracy (88.75%) compared to using a central cross displayed across the entire screen (74.25%). Interestingly, the most discriminative temporal features differed between the two conditions. In the case where the cue was presented on one side of the screen, the most informative features were localized around 50 ms before to 100 ms after cue onset. In contrast, for the condition with the cross displayed across the entire screen, the most discriminative features were found between 200 ms and 350 ms after the cue onset. These findings raise important questions about the influence of visual ERP elicited by the cue presentation used in typical MI-BCI paradigm, on classification performance.

To correctly evaluate the presence of bias in the learned model, many techniques exist, such as saliency maps or perturbation-based methods (Montavon et al., 2018; Nielsen et al., 2022). They are all gathered under the term “explainable artificial intelligence (XAI)”. See Tjoa and Guan (2021) for a survey on this topic. XAI tools can be used to evaluate the relevance and neurophysiological plausibility of the features extracted by the MI-BCI model. While some studies incorporated this type of analysis (Hartmann et al., 2018; Mane et al., 2021; Lawhern et al., 2018), it is not yet a standard practice (Tjoa and Guan, 2021). Nonetheless, comparing the features used by the model to known neurophysiological markers of the decoded mental task should be considered essential. This helps ensure that the model's decisions are not only accurate but also grounded in meaningful brain activity, thereby reducing bias and improving scientific validity.

Although the influence of visual cues may seem straightforward to part of the neuroscientific community, their specific effect on BCI classification accuracy has never been systematically investigated. As a result, various studies and BCI competitions, for example the NeurIPS 2021 BEETL Competition (Wei et al., 2022), include the visual cue within the epoching window, which may introduce bias into the reported results.

In this paper, we thus decided to evaluate the impact of including the time of the visual cue onsets, indicating the MI task to perform, in the input EEG epoching window and assess if it can induce a bias in the classification process with a deep learning approach (DeepConvNet Schirrmeister et al., 2017 and REVE El Ouahidi et al., 2025). We also compare those results with a more standard approach, CSP combined with LDA. This is particularly relevant to evaluate since deep learning approaches are able to filter the signal in the temporal and spatial dimension - and thus to extract temporal features - while CSP+LDA uses the mean of the spatially filtered signal power across the epoching window chosen, i.e., no temporal information. To compare the two models, we will evaluate them regarding two criteria, the model accuracy and the features learned by the model. The features learned by the model will be assessed using attribution maps for deep learning approaches and CSP patterns for CSP+LDA.

This article is divided as follows: first, in Section 2, we present the experimental setup, the dataset used and the different approaches that we used to train the models and present in detail the methodology of attribution maps. Then, in Section 3, we present the classification accuracy for each epoching condition (with and without visual cue onset) and each training condition (machine learning and deep learning). Lastly, in Section 4 we discuss the implications of those results, pointing out the necessity to not only rely on accuracy metrics but also to evaluate the neurophysiological features extracted.

## 2 Materials and methods

We investigated the impact of including the visual cue onset in the model input window on the performance of a convolutional neural network-based model, DeepConvNet (Schirrmeister et al., 2017), a Transformer-based EEG foundation model, REVE (El Ouahidi et al., 2025), and a widely used CSP+LDA model (Ramoser et al., 2000).

To this end, we first describe the open-source datasets used in this study (Dreyer et al., 2023; Lee et al., 2019) and the experimental protocol designed to evaluate the influence of including the visual cue onset in the input data. We then introduce the three learning approaches considered in this work, namely CSP+LDA, DeepConvNet, and REVE. Finally, we present the methods used to interpret the learned features, namely CSP patterns and attribution maps.

### 2.1 Dataset

Two open-access EEG datasets, Dreyer2023 and Lee2019, were used for this study. A Python library, MOABB (Mother of All BCI Benchmark) (Chevallier et al., 2024), was used to access these datasets.

#### 2.1.1 Dreyer2023 dataset

It contains data from 87 participants collected over a single day of MI-BCI experiments (Dreyer et al., 2023).

The experimental protocol was organized into a single session of MI, divided into six runs each lasting 7-min. Participants were required to engage in 20 MI tasks (trials) for each hand (left- and right-hand MI) per run. The first two runs corresponded to a calibration phase, where the feedback provided does not reflect the participants' actual MI performance, but was a sham feedback prerecorded from another subject and then presented to the participant. This approach allows the subject to perceive a feedback signal while performing the MI task, despite the feedback not being contingent on their own brain activity. Based on the EEG data from these two first runs, a linear classifier employing three pairs of CSP spatial filters and a LDA classifier was trained on the filtered data with band-pass filtered (5–35 Hz), see Dreyer et al. (2023) for more details. In the subsequent four runs, participants received real-time feedback based on the confidence of the classifier predictions.

During each run, users had to perform 40 trials, each trial lasted 8s. As described in Figure 1, at *t* = −3 s, a cross was displayed on the screen. At *t* = −1 s, an acoustic signal announced the appearance of a red arrow, which appeared one second later (at *t* = 0 s) and remained displayed for 1.25 s. The arrow pointed in the direction of the task to be performed (i.e., left for left-hand MI, right for right-hand MI). Participants were instructed to start performing the corresponding MI-task as soon as the arrow appeared, and to keep doing so until the cross disappeared. Finally, from *t* = 1.25 s, a visual feedback was continuously provided in the shape of a blue bar, the length of which varied proportionally to the BCI classifier output, i.e., the distance to the LDA hyperplane (for runs 3 to 6) or based on a sham signal (for the first two runs). The feedback was provided for 3.75 s and was updated at 16 Hz (1 s sliding window). After 8 s, the screen turned black again until the beginning of the next trial. The participant could then rest for a few seconds: from 1.5 s to 3.5 s, randomly chosen. Only positive feedback was displayed, i.e., the feedback was provided only when the recognized task matched the instructed task. The goal for participants was to maximize the length of this bar through their MI efforts. This visualization of the feedback is based on the Graz feedback training protocol (Pfurtscheller and Neuper, 2001). EEG signals were recorded with two g.USBAmp amplifiers (g.tec, Austria), sampled at 512 Hz, from 27 electrodes around the primary motor and the somatosensory cortices (*F*_*z*_, *F*_4_, *FC*_*z*_, *FC*_1_, *FC*_2_, *FC*_3_, *FC*_5_, *FC*_4_, *FC*_6_, *F*_3_, *C*_*z*_, *C*_5_, *C*_3_, *C*_1_, *C*_2_, *C*_4_, *C*_6_, *CP*_5_, *CP*_3_, *CP*_1_, *CP*_*z*_, *CP*_2_, *CP*_4_, *CP*_6_, *P*_*z*_, *P*_3_, *P*_4_).

**Figure 1:**
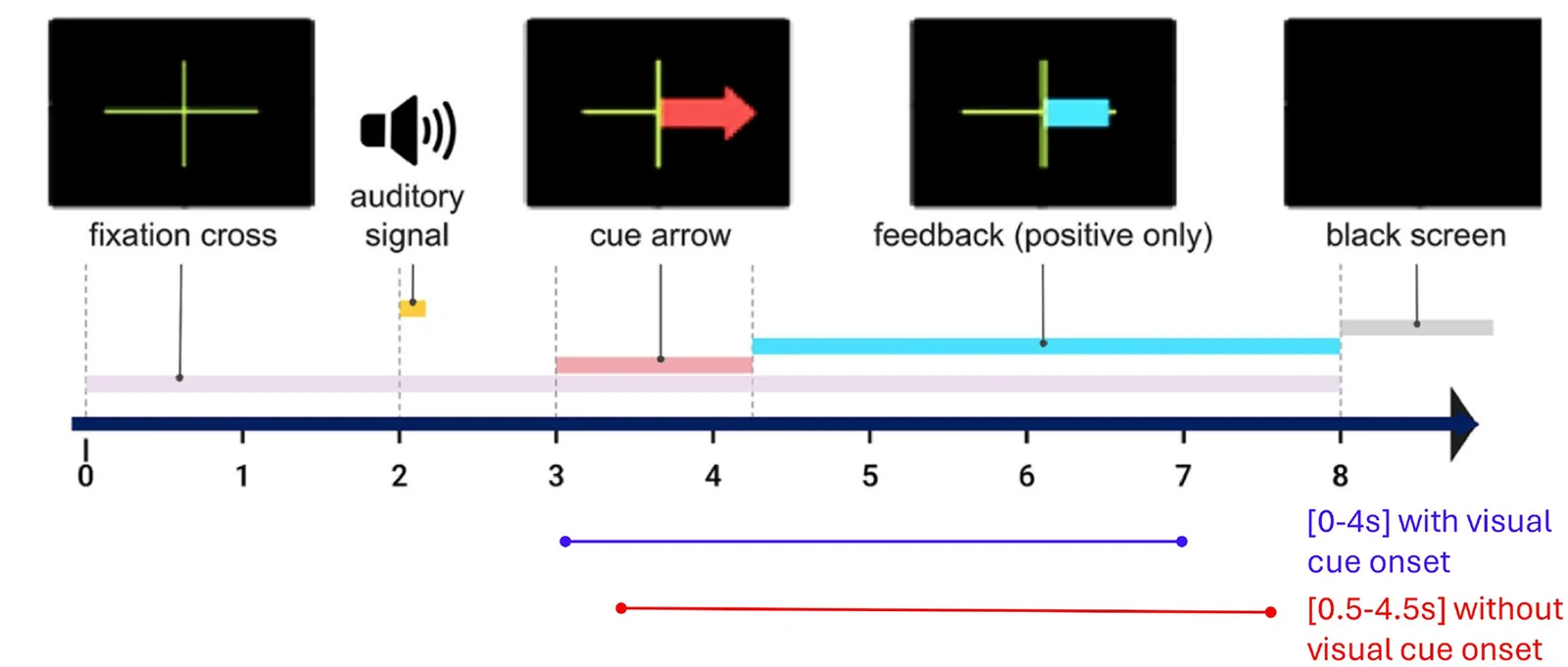
Illustration of the two windows evaluated for each trial, in the Dreyer2023 dataset for MI-BCI tasks using the Graz protocol. The blue and red windows correspond to the conditions with and without visual cue onset inclusion, respectively. See Dreyer et al. (2023) for further details.

The mean online classification accuracy across subjects reported by Dreyer et al. (2023) is 63.56% with a large variability between subjects, ranging from chance level (50%) to perfect classification accuracy (100%).

Two participants, identified as subjects 40 and 59, were excluded from the analysis due to missing trials that could potentially impact the data analysis pipeline in subsequent steps. This left 85 subjects for the analyses reported in the present paper.

#### 2.1.2 Lee2019 dataset

This dataset contains data from 54 participants collected over two days (sessions) of MI-BCI experiments (Lee et al., 2019). In each session, two runs (a training run and a test run) of MI-BCI tasks involving left- and right-hand motor imagery were conducted. As the test runs do not include the ground-truth labels, only the training runs were used in this study. Each training run consisted of 100 trials (50 trials per class).

As shown in Figure 2, each trial lasted 7 s. At *t* = −3 s, a fixation cross was presented on the screen, and at *t* = 0 s, an arrow appeared to indicate the motor imagery task. The motor imagery task lasted for 4 s. EEG signals from 62 channels (*Fp*_1_, *Fp*_2_, *F*_7_, *F*_3_, *F*_*z*_, *F*_4_, *F*_8_, *FC*_5_, *FC*_1_, *FC*_2_, *FC*_6_, *T*_7_, *C*_3_, *C*_*z*_, *C*_4_, *T*_8_, *TP*_9_, *CP*_5_, *CP*_1_, *CP*_2_, *CP*_6_, *TP*_10_, *P*_7_, *P*_3_, *P*_*z*_, *P*_4_, *P*_8_, *PO*_9_, *O*_1_, *O*_*z*_, *O*_2_, *PO*_10_, *FC*_3_, *FC*_4_, *C*_5_, *C*_1_, *C*_2_, *C*_6_, *CP*_3_, *CP*_*z*_, *CP*_4_, *P*_1_, *P*_2_, *PO*_*z*_, *FT*_9_, *FTT*_9*h*_, *TTP*_7*h*_, *TP*_7_, *TPP*_9*h*_, *FT*_10_, *FTT*_10*h*_, *TPP*_8*h*_, *TP*_8_, *TPP*_10*h*_, *F*_9_, *F*_10_, *AF*_7_, *AF*_3_, *AF*_4_, *AF*_8_, *PO*_3_, *PO*_4_) were recorded at a sampling frequency of 1000 Hz. For further details, please refer to the original paper (Lee et al., 2019).

**Figure 2:**
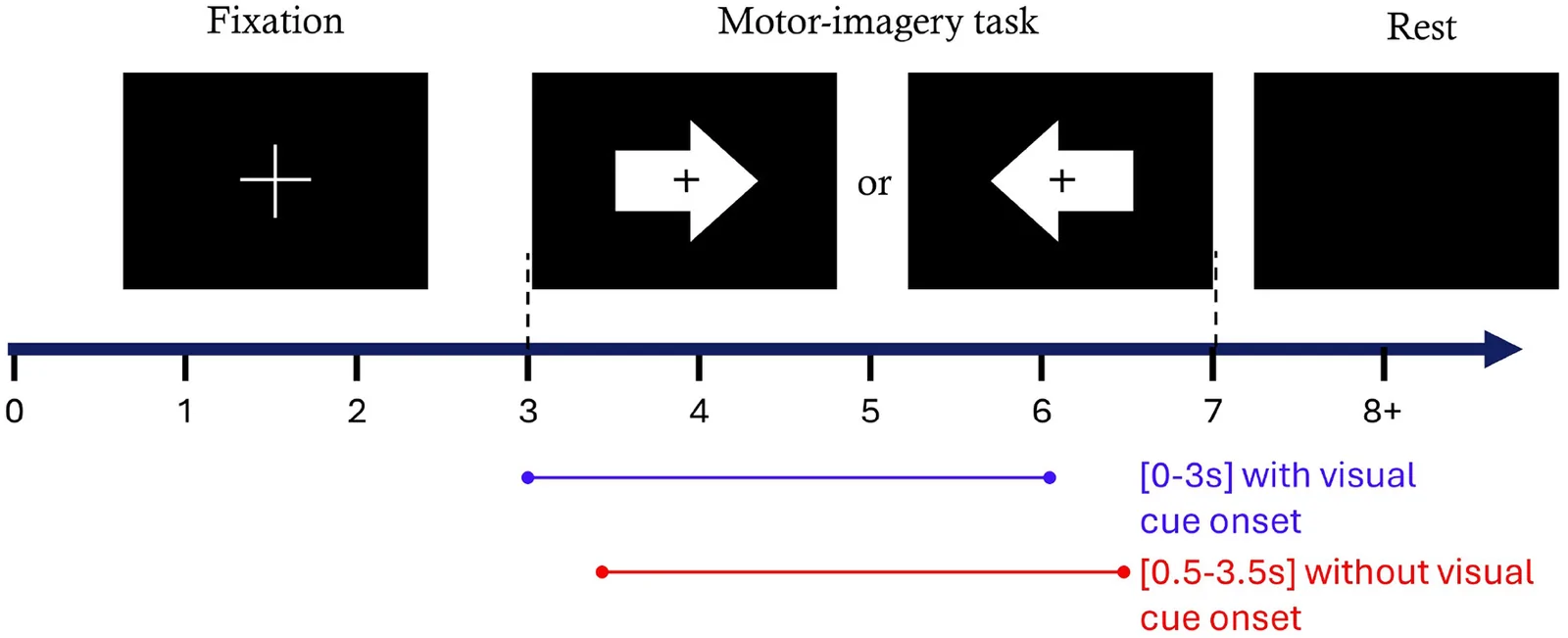
Illustration of the two input windows evaluated for each trial in the (Lee et al., 2019) dataset. The blue and red windows correspond to the conditions with and without visual cue onset inclusion, respectively. See Lee et al. (2019) for further details.

### 2.2 Experimental protocol

To evaluate the influence of including the visual cue onset in the input window, two epoch windows were considered for all models: 0.0–4.0 s and 0.5–4.5 s for the Dreyer2023 dataset, and 0.0–3.0 s and 0.5–3.5 s for the Lee2019 dataset. The windows were selected such that both conditions contained the same number of samples. Using the (Dreyer et al., 2023) dataset as an example, Figure 1 illustrates the two epoch windows examined in this study. The first window began at cue onset (0.0–4.0 s), whereas the second was shifted by 0.5 s (0.5–4.5 s), thereby excluding the visual cue onset. Those two conditions will be referred to as epoching including visual cue onset and epoching window without visual cue onset, respectively, as we consider that mental processing due to the appearance of the visual cue is shorter than 500 ms (Nehamkin et al., 2008; Pfurtscheller et al., 2013). All other preprocessing and training parameters were kept identical across conditions to ensure a controlled comparison of the effects of visual cue onset inclusion on model performance and the extracted features.

We evaluated three classification pipelines: a traditional Common Spatial Patterns and Linear Discriminant Analysis (CSP+LDA) approach, DeepConvNet, a convolutional neural network-based architecture (Schirrmeister et al., 2017), and REVE, a Transformer-based EEG foundation model (El Ouahidi et al., 2025). Since CSP+LDA is primarily used in within-subject BCI settings (Lotte et al., 2018), whereas deep learning models have demonstrated strong cross-subject generalization capabilities (Kwon et al., 2019), we evaluated CSP+LDA in the within-subject setting and DeepConvNet and REVE in the cross-subject setting. For direct comparison, CSP+LDA was also evaluated in the cross-subject setting.

In the cross-subject paradigm, we employed a leave-one-subject-out strategy: each subject was used once as the test set, while the remaining subjects served as the training set. For DeepConvNet and REVE, an additional validation split was performed on the training subjects: 80% for training and 20% for validation.

For the Dreyer2023 dataset, all runs (1–6) from each training subject were included in the training set, whereas only the online feedback runs (3–6) were used for the validation and test subjects. For the within-subject paradigm, models were trained on the two initial calibration runs and evaluated on the four subsequent online feedback runs.

For the Lee2019 dataset, all trials from the training runs of the training subjects (100 trials in total, 50 per class) were included in the training set. For evaluation, the last 25 trials from each class (50 trials in total) of the validation and test subjects were used for validation and testing. For the within-subject paradigm, the first 25 trials from each class were used for training, whereas the remaining 25 trials per class were used for testing.

To evaluate the influence of removing EEG channels associated with visual responses, an additional analysis was conducted using the Dreyer2023 dataset and DeepConvNet. In this analysis, the parietal channels (*P*3, *P*_*z*_, and *P*4) were excluded. All other experimental procedures and parameters were identical to those used in the other conditions.

### 2.3 Neurophysiological features of MI

As the users were performing left- and right-hand MI, the targeted neurophysiological features are the event-related desynchronization (ERD) and event-related synchronization (ERS) characteristics of motor imagery and motor execution. The ERD corresponds to a decrease of EEG power, within the μ band (8-13 Hz) and β band (13-30 Hz) of the contralateral sensory motor cortex during the movement execution/imagination (Kilavik et al., 2013), followed by an ERS, i.e an augmentation of the EEG signal power within the β band after the executed/imagined movement ends. This pattern was first introduced by Pfurtscheller and Lopes da Silva (1999). The ERD/ERS patterns exhibit both temporal and spatial characteristics. Temporally, the ERD typically emerges at the onset of motor imagery and persists throughout the duration of the task, e.g., until *t* = 8s for the Dreyer2023 dataset. This is usually followed by an ERS phase after the imagery ends. Spatially, the ERD/ERS activity during left- and right-hand MI is localized around the C3 and C4 electrodes, with contralateral correspondence: ERD/ERS in the right hemisphere is associated with the execution or imagination of movements involving the left hand, and vice versa. Research has demonstrated that this pattern can enable the creation of reliable BCIs, with accuracies around 77% (Neuper et al., 2005). Moreover, the ERD/ERS pattern has been shown to exhibit strong consistency and stability over time (Neuper et al., 2005). Importantly, the strength and location of ERD/ERS patterns can vary between individuals, reflecting differences in brain anatomy, cognitive strategies, and experience with motor imagery tasks, which can impact the individual's ability to control a BCI (from chance level to 100% of accuracy Dreyer et al., 2023).

### 2.4 Deep learning training

#### 2.4.1 DeepConvNet

To evaluate the influence of the choice of the epoching window in deep learning model we trained the two different window conditions with the DeepConvNet model initially proposed by Schirrmeister et al. (2017). DeepConvNet consists of four convolutional blocks and one classification block. Each convolutional block includes a convolutional layer to capture specific patterns in EEG signals and a max-pooling layer to reduce dimensionality and variability. The first block slightly differs as it contains two convolutional layers: a temporal layer that applies kernels along the time dimension to capture frequency-specific patterns and a spatial layer that applies kernels across all channels to capture interactions between channels. The final layer is a classification layer, with a dense layer that can classify multiple classes (e.g., four in the original study, though it can be adapted for binary classification). Due to its depth, this model requires extensive training data to optimize its 283,202 parameters for a 4-second trial (see Table 1). Consequently, DeepConvNet is often employed in cross-subject training scenarios where a larger number of trials is thus available for training (combining the trials from multiple subjects) (Lawhern et al., 2018).

**Table 1:** Detailed architecture of the DeepConvNet model.

Layer (type)	Output shape	Parameters
Ensure4d	[−1, 27, 1000, 1]	0
Expression	[−1, 1, 1000, 27]	0
Conv2d (Temporal)	[−1, 25, 991, 27]	275
Conv2d (Spatial)	[−1, 25, 991, 1]	16,875
BatchNorm2d	[−1, 25, 991, 1]	50
Expression	[−1, 25, 991, 1]	0
MaxPool2d	[−1, 25, 330, 1]	0
Expression	[−1, 25, 330, 1]	0
Dropout	[−1, 25, 330, 1]	0
Conv2d	[−1, 50, 321, 1]	12,500
BatchNorm2d	[−1, 50, 321, 1]	100
Expression	[−1, 50, 321, 1]	0
MaxPool2d	[−1, 50, 107, 1]	0
Expression	[−1, 50, 107, 1]	0
Dropout	[−1, 50, 107, 1]	0
Conv2d	[−1, 100, 98, 1]	50,000
BatchNorm2d	[−1, 100, 98, 1]	200
Expression	[−1, 100, 98, 1]	0
MaxPool2d	[−1, 100, 32, 1]	0
Expression	[−1, 100, 32, 1]	0
Dropout	[−1, 100, 32, 1]	0
Conv2d	[−1, 200, 23, 1]	200,000
BatchNorm2d	[−1, 200, 23, 1]	400
Expression	[−1, 200, 23, 1]	0
MaxPool2d	[−1, 200, 7, 1]	0
Expression	[−1, 200, 7, 1]	0
Conv2d (Classifier)	[−1, 2, 1, 1]	2,802
LogSoftmax	[−1, 2, 1, 1]	0
Expression	[−1, 2]	0

The input to the network has a shape of [27, 1, 000], where 27 corresponds to EEG channels and 1,000 to time samples. All convolutional and pooling operations are applied with appropriate padding to maintain valid dimensions.

All input data for DeepConvNet were normalized using the mean and standard deviation computed from the training set. Training followed the standard hyperparameters proposed by Schirrmeister et al. (2017) with a batch size of 256 and 150 epochs. The detailed architecture of the table is synthesized in Table 1. For this experiment, we temporally filtered the signal between 0.5Hz and 40Hz and downsampled to 250 Hz, we did not reject trials based on the presence of artifacts. The best-performing model across training iterations, based on validation performance, was saved and subsequently evaluated on the test set and used for the evaluation of the attribution maps.

#### 2.4.2 REVE

REVE is a state-of-the-art EEG foundation model that has outperformed conventional deep learning models as well as other EEG foundation models (El Ouahidi et al., 2025). The pretrained model was trained on more than 6,000 hours of EEG recordings from over 25,000 subjects, making REVE the largest pretrained EEG foundation model to date. REVE is based on the Transformer architecture, and in this study, we used the *base* model, which contains 69,410,206 learnable parameters.

To leverage the pretrained parameters, we adopted a preprocessing pipeline similar to that used by the original authors. Specifically, the EEG signals were bandpass filtered between 0.5 and 40 Hz, downsampled to 200 Hz, and normalized using the mean and standard deviation computed from the training set. Samples exceeding 15 times the standard deviation of the training set were clipped. During training, a batch size of 256 was used. The final classification layer was first trained while keeping the pretrained backbone frozen (linear probing), after which all model parameters were optimized during fine-tuning. For linear probing, training was performed for up to 200 epochs with early stopping, using a warm-up period of 5 epochs and a patience of 15 epochs. Fine-tuning was then performed for 5 epochs. Validation accuracy was monitored throughout training, and the model with the highest validation accuracy was retained for evaluation and attribution analysis.

#### 2.4.3 Attribution maps

Deep learning models, despite their strong performance in various classification tasks, are often criticized for their lack of interpretability. This opacity, commonly referred to as the “black box” problem (Tjoa and Guan, 2021), makes it challenging to understand which features or patterns in the input data drive the model's decisions. To mitigate this black-box effect, various explainability techniques have been proposed.

One class of explainability techniques aims to identify which parts of the input contribute most strongly to a model's prediction. These methods generate attribution maps that visualize the contribution of each input feature to the model output (Nielsen et al., 2022; Montavon et al., 2018). In this study, we employed three widely used attribution methods: Saliency Maps (Simonyan et al., 2014), Integrated Gradients (Sundararajan et al., 2017), and DeepLIFT (Shrikumar et al., 2017). In the context of BCI, attribution maps can help assess whether the features a model uses are neurophysiologically meaningful. In this work, we applied attribution methods to DeepConvNet and REVE to better understand the spatiotemporal features these models use to discriminate between left- and right-hand MI. For each test epoch, represented as an input matrix of size ℝ^*C*×*T*^ (*C*: number of EEG channels, *T*: number of time samples), attribution methods produced an attribution map of the same size. To facilitate interpretation, we transformed each attribution map into two complementary representations: a temporal attribution vector (ℝ^*T*^) and a spatial attribution vector (ℝ^*C*^). These representations were obtained by averaging attribution scores across channels or time samples, respectively. Finally, attribution scores were averaged across test trials and subjects to obtain a single temporal and spatial attribution profile for each model.

### 2.5 CSP+LDA training

The combination of CSP and LDA is the most widely used algorithm for online BCI application (Lotte et al., 2018). The CSP+LDA pipeline consists of two steps: First a spatial filtering step that projects EEG signals onto a subspace where class-discriminative information is maximized, and second a classification step, performed with LDA, that assigns the filtered signals to a class label. CSP filters maximize the variance of the spatially filtered signal under one condition while minimizing it for the other condition (Blankertz et al., 2008). LDA performs a linear projection of the input data vector, here the log variance of the EEG signals spatially filtered with CSP, with optimized weights to minimize within-class variance while maximizing between-class variance. It has been originally proposed by Fisher (1936). This two step optimization, improves class separability, providing robust feature representations for classification of MI-tasks. The CSP+LDA equation can be written as follows Equation 1:


$$\begin{array}{c} f(\text{X};{\left\{{\text{w}}_{\text{j}}\right\}}_{j=1}^{J},{\left\{{\text{a}}_{\text{j}}\right\}}_{j=1}^{J})=\sum_{j=1}^{J}{\text{a}}_{\text{j}}\cdot \log\left({\text{w}}_{\text{j}}^{⊤}\text{X}{\text{X}}^{⊤}{\text{w}}_{\text{j}}\right)+\beta_{0} \end{array}$$ (1)

In this equation, **X** denotes the input matrix with the dimension of ℝ^*C*×*T*^. The terms *a*_*j*_ (with *j* ∈ {1, …, *J*}) and β_0_ represent the weights and the intercept of the LDA, respectively, while *w*_*j*_ (with *j* ∈ {1, …, *J*}) denote the spatial filters obtained with the CSP algorithm. The variable *J* refers to the number of spatial filters, here *J* = 6 since we used (as typical) three pairs of filters.

For the CSP+LDA pipeline, we temporarily filtered the signal between 8Hz and 30Hz.

#### 2.5.1 CSP patterns

CSP filters maximize the variance of the spatially filtered signal under one condition while minimizing it for the other condition. They work as pairs, where one filter maximizes variance for one condition, here left-hand MI, and the second filter maximizes it for the other condition, here right-hand MI. Yet, spatial filters are not easily interpretable, they are a combination of weights for each electrode, such weight possibly reflecting both task-related importance but also noise to filter out, which makes visual interpretation difficult. From the spatial filter **w**, for interpretability, one should rather compute the corresponding spatial pattern **a** (see Equation 2), which illustrates how the presumed neural sources extracted by the spatial filter project onto the scalp (Blankertz et al., 2008). This enables a neurophysiological validation of the filter's plausibility. Here we except that the features extracted are located in the sensory motor cortex as it should be for SMR during MI.


$$\begin{array}{c} a={\left(w^{-1}\right)}^{⊤} \end{array}$$ (2)

### 2.6 Statistical analysis

This study evaluated the effect of visual cues on classification performance. For the deep learning approaches, we analyzed two models, DeepConvNet and REVE, using two datasets, Dreyer2023 and Lee2019. Since the presence and absence of visual cue onset in the epoching window were compared for each combination of model and dataset, a total of four comparisons (2 models × 2 datasets) were performed. For the conventional approach, CSP+LDA, we also analyzed the Dreyer2023 and Lee2019 datasets using both within-subject and cross-subject evaluation schemes. Consequently, four comparisons (2 datasets × 2 evaluation schemes) were conducted to assess the effect of visual cues.

To evaluate the statistical significance of the performance differences between conditions with and without visual cue onset, two-sided paired t-tests were performed for each comparison. In total, eight statistical tests were conducted, comprising four comparisons for the deep learning approaches and four comparisons for CSP+LDA. Since a total of eight statistical tests were conducted, the resulting p-values were corrected for multiple comparisons using the Benjamini–Hochberg procedure (Benjamini and Hochberg, 1995).

### 2.7 Visual ERP analysis

In addition to the machine learning and attribution analyses, we performed an Event Related Potential (ERP) analysis, in order to investigate the time-locked effect of the visual cue onset on EEG signals, in particuler on ERPs. First, the EEG data were band-pass filtered between 0.5 and 40 Hz. After filtering, the Lee2019 dataset was downsampled to 512 Hz. Epochs were extracted from −3.5 to 5.5 s relative to the onset of the visual cue, and baseline correction was performed using the interval from −0.8 to −0.2 s. Epochs in which the EEG amplitude exceeded ±150μV during the task period (Dreyer2023: 0.5–4.5 s; Lee2019: 0.5–3.5 s) were excluded from further analysis. For each subject, the remaining epochs were averaged across trials separately for each class to obtain subject-level ERPs. These subject-level ERPs were then averaged across subjects to obtain the grand-average ERP.

Furthermore, to quantify the separability between the left- and right-hand classes, the signed-*r*^2^ metric (Blankertz et al., 2011) was computed for each EEG channel, with positive values indicating larger EEG amplitudes for the left-hand class than for the right-hand class.

The ERP analysis was performed for three groups. First, all subjects in each dataset were included, in order to investigate the overall ERP effect of the visual cue onset. The remaining two groups consisted of subjects who exhibited either a large or a small change in classification accuracy when the visual cue onset was included in the decoding window compared with when it was excluded. This enabled us to study inter-subjects variability in the visual cue onset effect, notably to study whether the ERPs differed between subjects for which removing the cue changed their classification accuracy the most and subjects for which removing it made the least change in their classification accuracy. Specifically, based on the DeepConvNet results (left panel of Figure 3), subjects were split into two groups using the median change in classification accuracy between decoding windows that included and excluded the visual cue onset. Subjects above the median were assigned to the large-difference group, whereas those below the median were assigned to the small-difference group. The same ERP analysis was then performed for each group.

**Figure 3:**
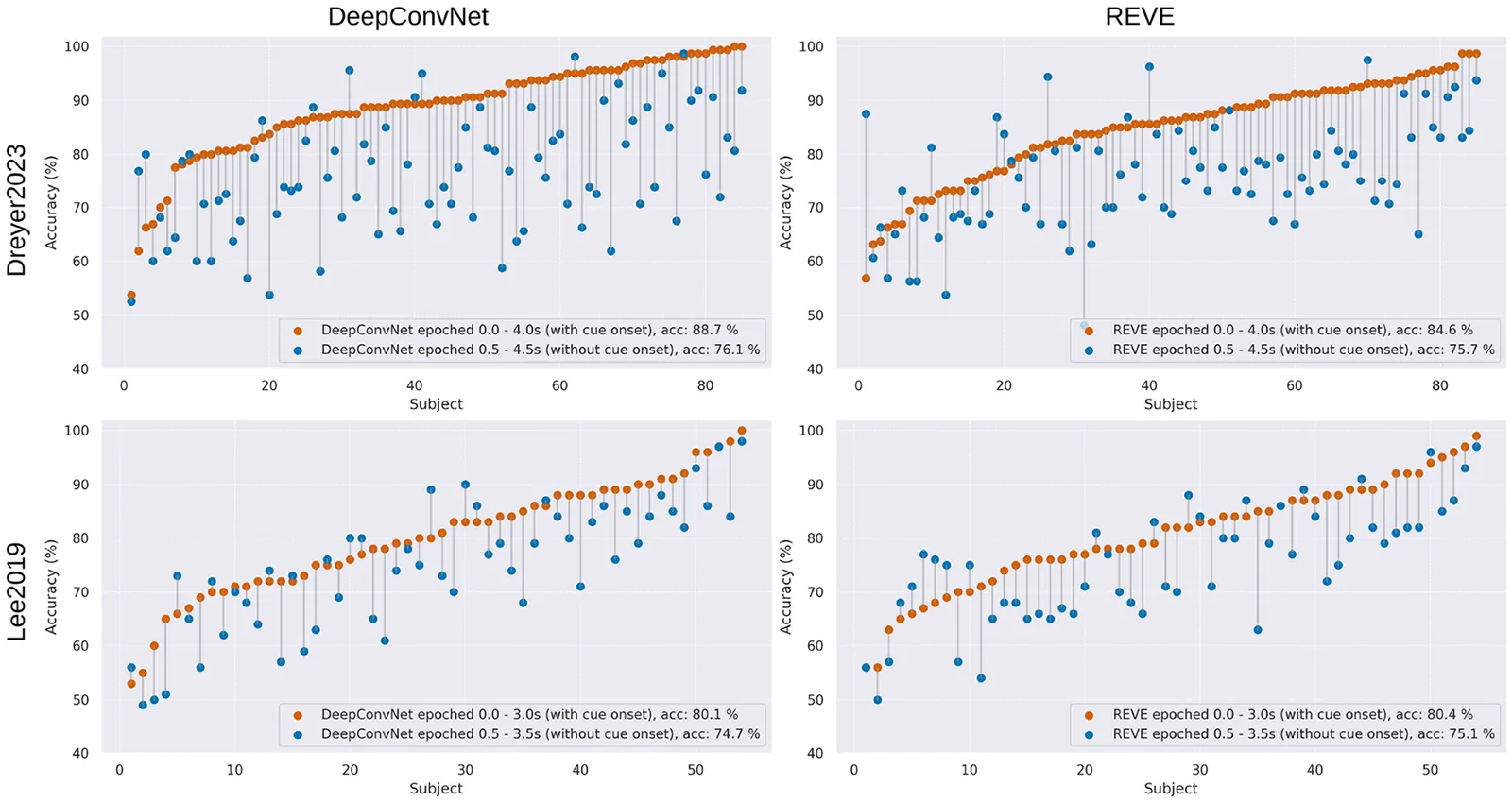
Accuracy of the DeepConvNet and REVE, for the cross-subject condition, for each subject with the two datasets and two different epoching approaches, with and without visual cue onset in the epoching window, respectively in orange and blue. Subjects are ordered by classification accuracy in the condition with visual cue onset.

## 3 Results

### 3.1 Classification accuracy and corresponding attribution maps for deep learning models

We trained the DeepConvNet and REVE using a cross-subject approach under two different epoching conditions. The classification results are summarized in Figure 3. The inclusion of visual cue onset consistently improved classification performance across both deep learning models and datasets. For DeepConvNet, accuracy increased by 12.6 % on Dreyer2023 and 5.4% on Lee2019. For REVE, the corresponding improvements were 8.9% and 5.3%, respectively. These improvements were statistically significant in all cases (DeepConvNet: *p* = 7.0 × 10^−17^ and *p* = 3.5 × 10^−7^; REVE: *p* = 1.0 × 10^−12^ and *p* = 1.9 × 10^−6^ for Dreyer2023 and Lee2019, respectively). This performance gain is consistent across subjects, with the model achieving its best performance for the majority of subjects when the visual cue onset is included. Therefore, we can conclude that including a visual cue does influence the classification accuracy.

The results obtained after removing the parietal channels from the Dreyer2023 dataset using DeepConvNet are shown in Supplementary Figure S3. Even when removing these paretial channels (i.e., the channels the most likely to capture visual cue processing EEG patterns in that data set), including the visual cue onset still improved the classification performance by 9.2% (*p* = 7.6 × 10^−15^) as compared to not including the visual cue onset (with the cue onset: 84.3%, without the cue: 75.1%). Thus, removing EEG channels related to visual processing is not sufficient to remove the influence of the visual cue.

In order to interpret the observed changes in classification accuracy, we used temporal and spatial attribution maps to analyze which features were exploited by the models. The saliency maps are illustrated in Figure 4. The Integrated Gradients and DeepLIFT attribution maps are shown in Supplementary Figures S1, S2, respectively. The attribution maps showed similar patterns across the three methods, with only minor differences.

**Figure 4:**
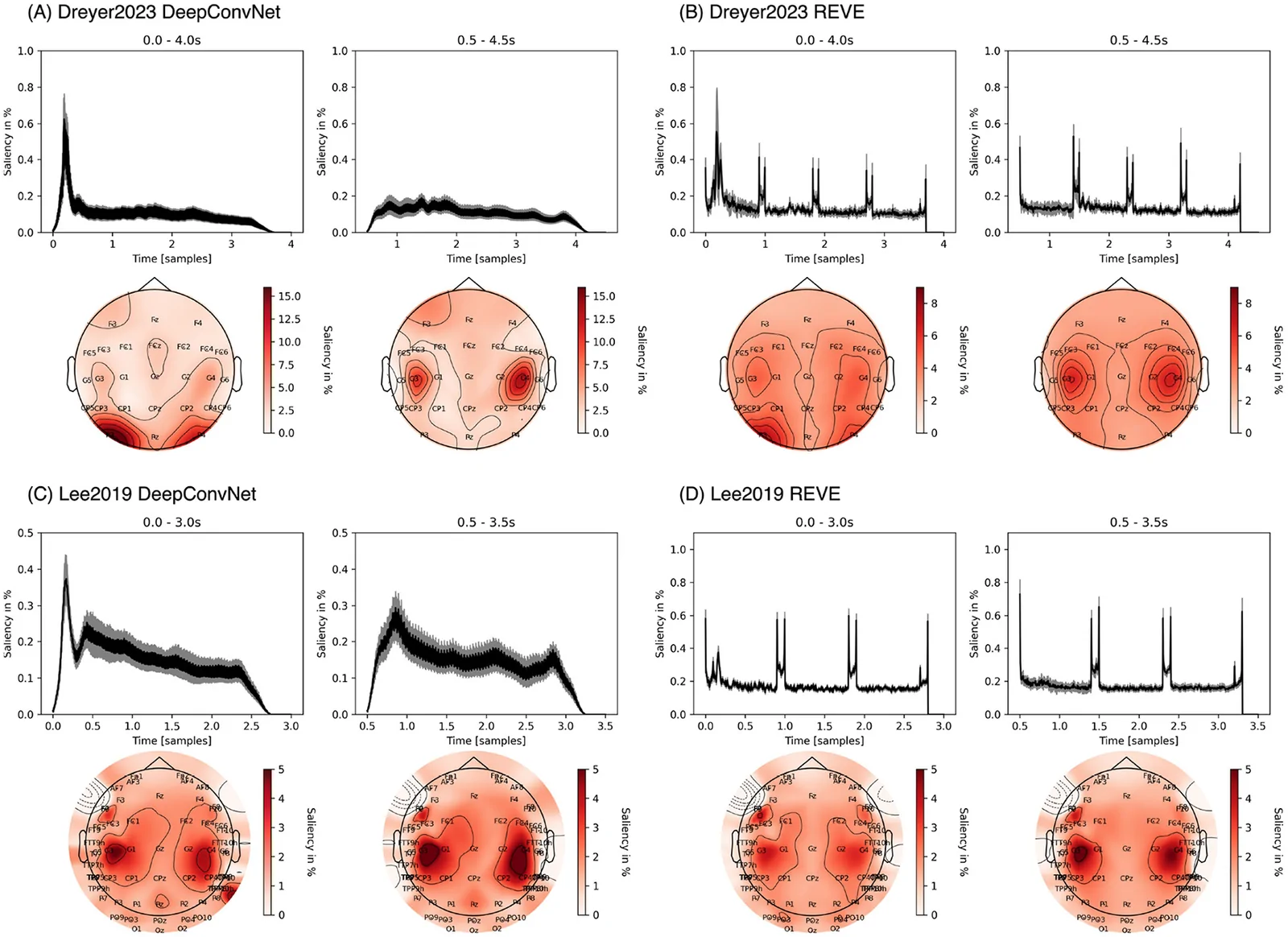
Temporal and spatial saliency maps for cross-subject training with DeepConvNet and REVE. **(A)** Dreyer2023 with DeepConvNet, **(B)** Dreyer2023 with REVE, **(C)** Lee2019 with Dee-pConvNet, and **(D)** Lee2019 with REVE. For each subfigure, the left panel shows the attribution maps obtained in the condition with the onset, whereas the right panel shows the attribution maps obtained in the condition without the cue onset. For the temporal attribution maps, the black line indicates the mean attribution across subjects, and the gray shaded area indicates the standard deviation. For the spatial attribution maps, red regions indicate stronger attribution values.

For the Dreyer2023 dataset, the attribution maps of both DeepConvNet and REVE indicated that the most discriminative information was concentrated within the first 0.5 s after cue onset. In addition, the most informative electrodes were located over the parietal regions, particularly around P3 and P4.

For the Lee2019 dataset, the temporal attribution maps obtained from DeepConvNet suggest that the model relies more heavily on information from approximately 0–0.5 s when the visual cue is present. Similarly, the temporal attribution maps of REVE exhibit a prominent peak around 0.2–0.3 s in the condition with the cue onset, whereas no corresponding peak is observed in the condition without the cue onset. Moreover, the spatial attribution maps indicate that both models place greater emphasis on information from regions surrounding the motor cortex, including C3 and C4, in the condition without the cue onset than in the condition with the cue onset.

These suggest that DeepConvNet and REVE may be using the brain response to cue presentation for classification, e.g., a visual evoked potential induced by the arrow's appearance or a neural correlate of visual attention orientation toward the arrow, to discriminate between left-hand and right-hand motor imagery trials.

In contrast, for the model trained without the visual cue onset included in the input window, the temporal saliency map reveals that discriminative information is distributed across the entire trial. Regarding the spatial saliency, the most discriminative electrodes are C3 and C4. The features extracted in this case resemble ERD-related patterns, originating from the sensorimotor cortex and persisting throughout the trial. Therefore, we can conclude that this model uses ERD features to discriminate between left-hand and right-hand motor imagery trials, as expected from the corresponding neuroscience literature (Nehamkin et al., 2008).

For the REVE attribution maps, temporal attribution peaks were observed at approximately 1 s intervals across all attribution methods. One possible explanation is the segmentation strategy employed by REVE, which divides the EEG signal into 1.0 s windows (El Ouahidi et al., 2025).

By quantitatively analyzing temporal and saliency profiles, we observe that the model trained with visual cue information is more specialized than the one relying solely on ERD-related features. In the condition with the visual cue onset (Dreyer2023 dataset with DeepConvNet), electrodes P4 and P3 account for 12.0% and 18.2% of the model's decision, respectively. In contrast, when the visual cue onset is excluded, electrodes C4 and C3 contribute only 13.0% and 10.2%, respectively. Similarly, in the temporal domain, the peak of discriminative information occurs around 0.19 seconds after stimulus onset in the condition with the visual cue onset, reaching a maximum contribution of 0.62% at a single time step. In contrast, in the condition without the visual cue onset, the discriminative peaks are smaller, around 0.19%, and spread over a longer time period.

### 3.2 CSP+LDA classification accuracy and corresponding CSP patterns

We trained the CSP algorithm combined with LDA using both within-subject and cross-subject approaches under two different epoching conditions. The classification results are summarized in Figure 5 for the within-subject approach and in Figure 6 for the cross-subject approach.

**Figure 5:**
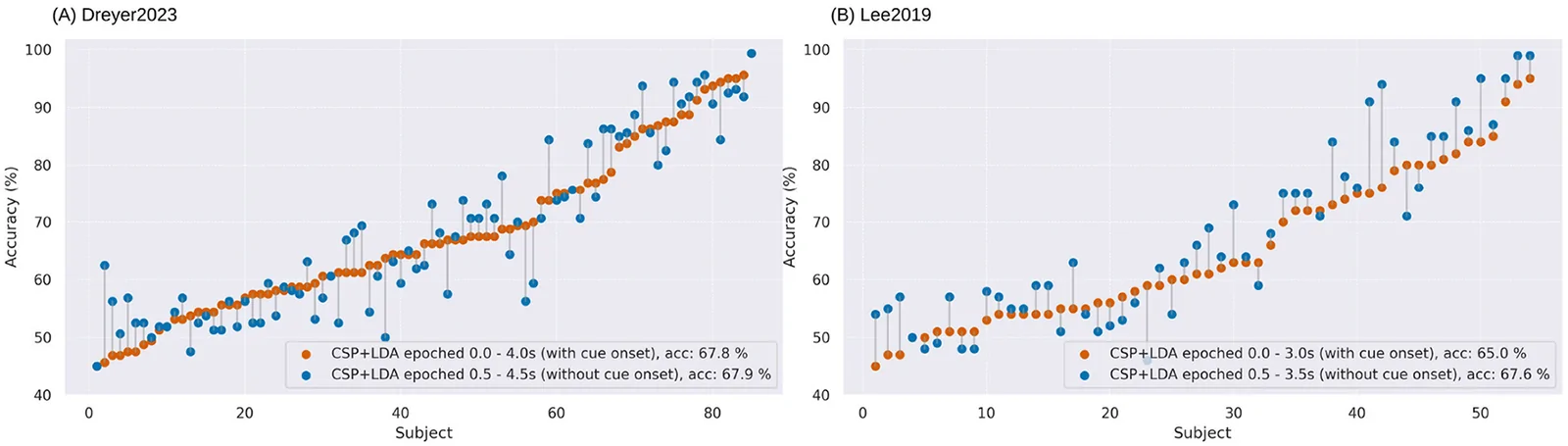
Accuracy of the CSP+LDA for within-subject training for each subject with the two different epoching approaches, with and without a visual cue onset in the epoching window, respectively red orange and blue. Subjects are ordered by classification accuracy in the condition with visual cue onset.

**Figure 6:**
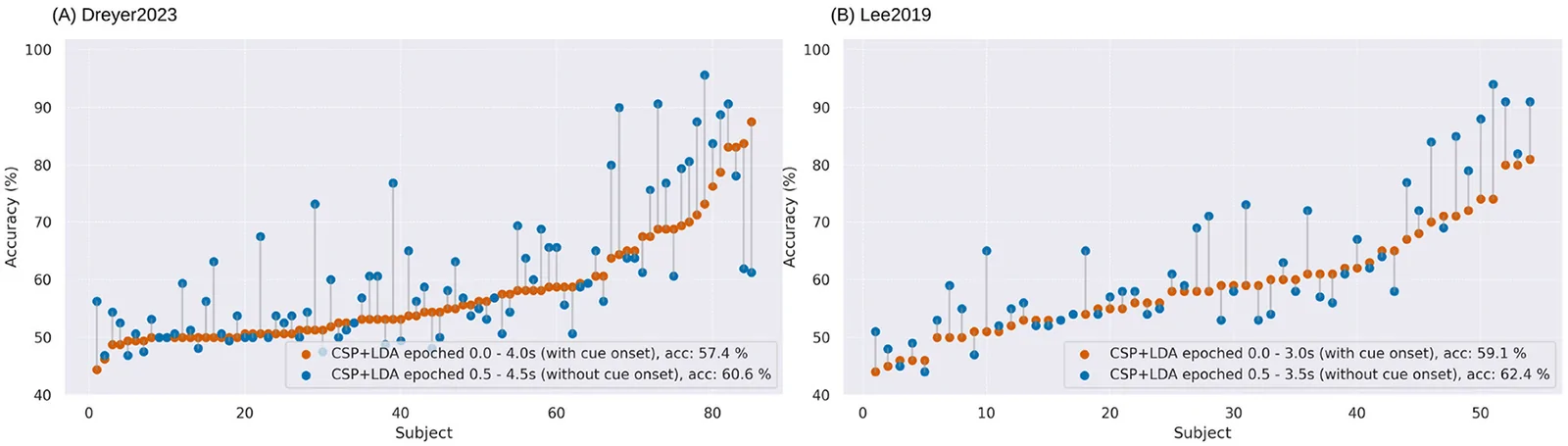
Accuracy of the CSP+LDA for cross-subject training for each subject with the two different epoching approaches, with and without a visual cue in the epoching window, respectively red orange and blue. Subjects are ordered by classification accuracy in the condition with visual cue onset.

In the within-subject approach, classification accuracies were almost identical (and with no significant differences) for both conditions, with and without the inclusion of the visual cue onset, with mean accuracies of 67.8% and 67.9% for Dreyer2023 (*p* = 0.77), respectively. For Lee2019, a minor yet statistically significant difference was observed, with mean accuracies of 65.0% and 67.6%, respectively (*p* = 2.2 × 10^−3^). However, despite the similarity in mean performance, individual subject results vary significantly. Some participants performed better when the model was trained without the visual cue onset, while others benefited from its inclusion. For some subjects, this difference reached up to 13.7%.

In the cross-subject approach, excluding the visual cue onset from the epoching window resulted in a significantly higher average classification accuracy for both datasets, although the effect size was modest (Dreyer2023: 60.6% vs. 57.4%, *p* = 1.6 × 10^−3^; Lee2019: 62.4% vs. 59.1%, *p* = 5.5 × 10^−4^). Most subjects achieved better performance in the condition without the visual cue onset. These findings contrast with those from the deep learning-based cross-subject approach, where including the visual cue onset resulted in improved classification accuracy.

Observing the CSP patterns extracted from the within-subject training (Figure 7), we notice that the patterns are very similar across the two conditions for most subjects. This supports the hypothesis that the features extracted by the model are largely equivalent in both conditions. However, for some subjects, such as subject 12, we observe notable differences. In the condition with the visual cue onset, two patterns (CSP0 and CSP2) clearly extract features from the parietal electrodes P3 and P4. In contrast, in the condition without the visual cue onset included in the epoching window, the corresponding CSP2 pattern differs significantly and does not reflect activity from the parietal areas.

**Figure 7:**
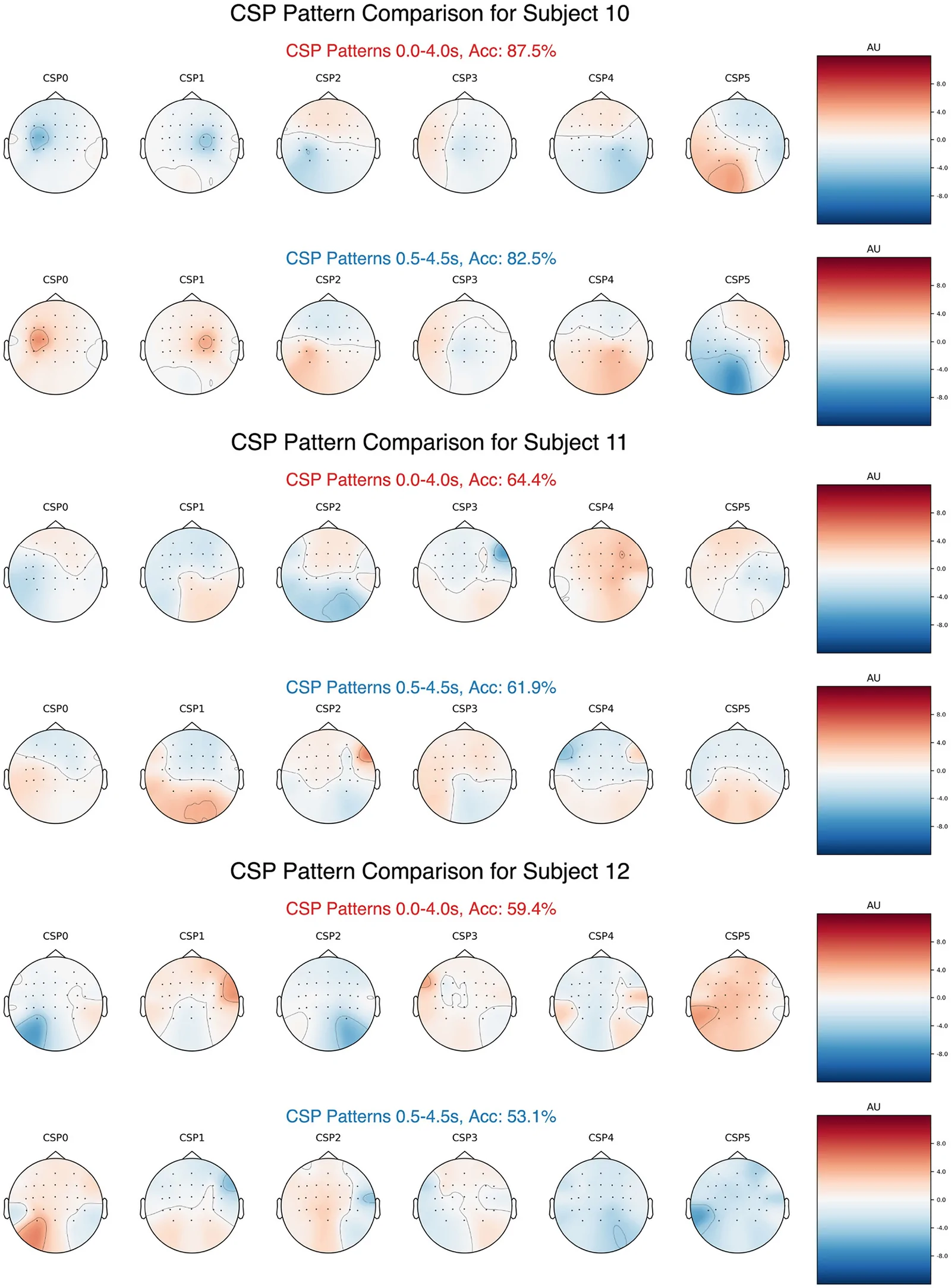
CSP patterns for three subjects trained in a within-subject approach with (epoching 0–4 s) and without visual cue onset (epoching 0.5–4.5 s) included in the four-second epoching window. All subjects are from the Dreyer2023 dataset.

While the extracted patterns appear similar between conditions in the within-subject setting, they differ markedly in the cross-subject setting, as illustrated in Figure 8. In cross-subject setting for both condition there is no clear and strong patterns associated with sensorimotor activity, corresponding to left- and right-hand motor regions. In the condition without the visual cue onset, two CSP patterns (CSP2 and CSP4) appears to have small patterns related to sensorimotor activity. In the condition with the visual cue onset, CSP pattern is slightly related to sensorimotor activity.

**Figure 8:**
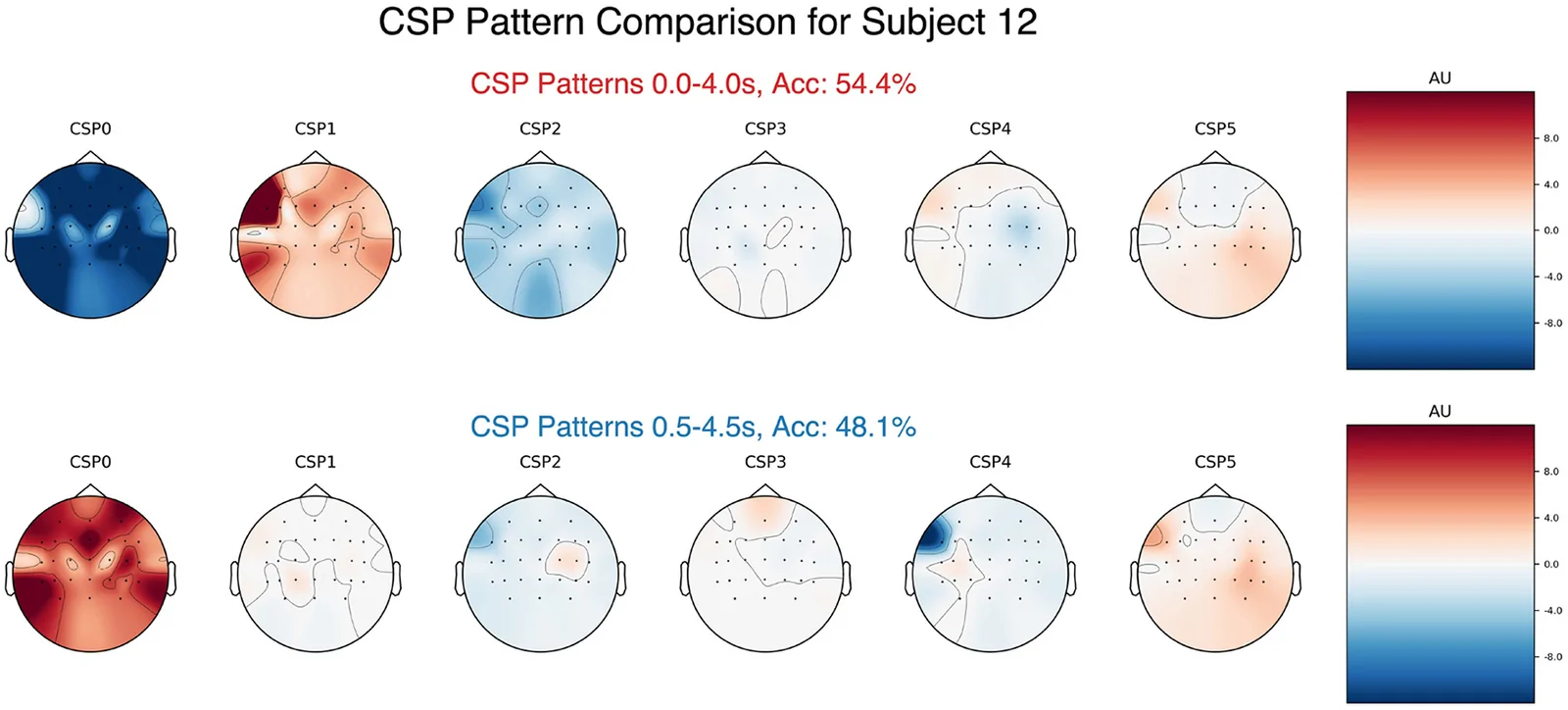
CSP patterns trained for the cross-subject classification of subject 12 in Dreyer2023 dataset. The training is done with (epoching 0–4 s) and without (epoching 0.5–4.5 s) visual cue onset in the four-second epoching window. As the training is made in a leave-one-out approach in a cross-subject, these patterns are extracted from the training of all subjects except the one tested. As the patterns are similar for all tested subjects, we visualize only one subject.

### 3.3 Visual ERP

Figures 9, 10 show the ERP responses and signed-r^2^ values for Dreyer2023 and Lee2019 datasets, respectively. In both datasets, the visual evoked potentials (VEPs) are observed around 0.1–0.2 s, and the central-parietal oriented positivity was observed around 0.3–0.5 s which is most likely to be P300 component.

**Figure 9:**
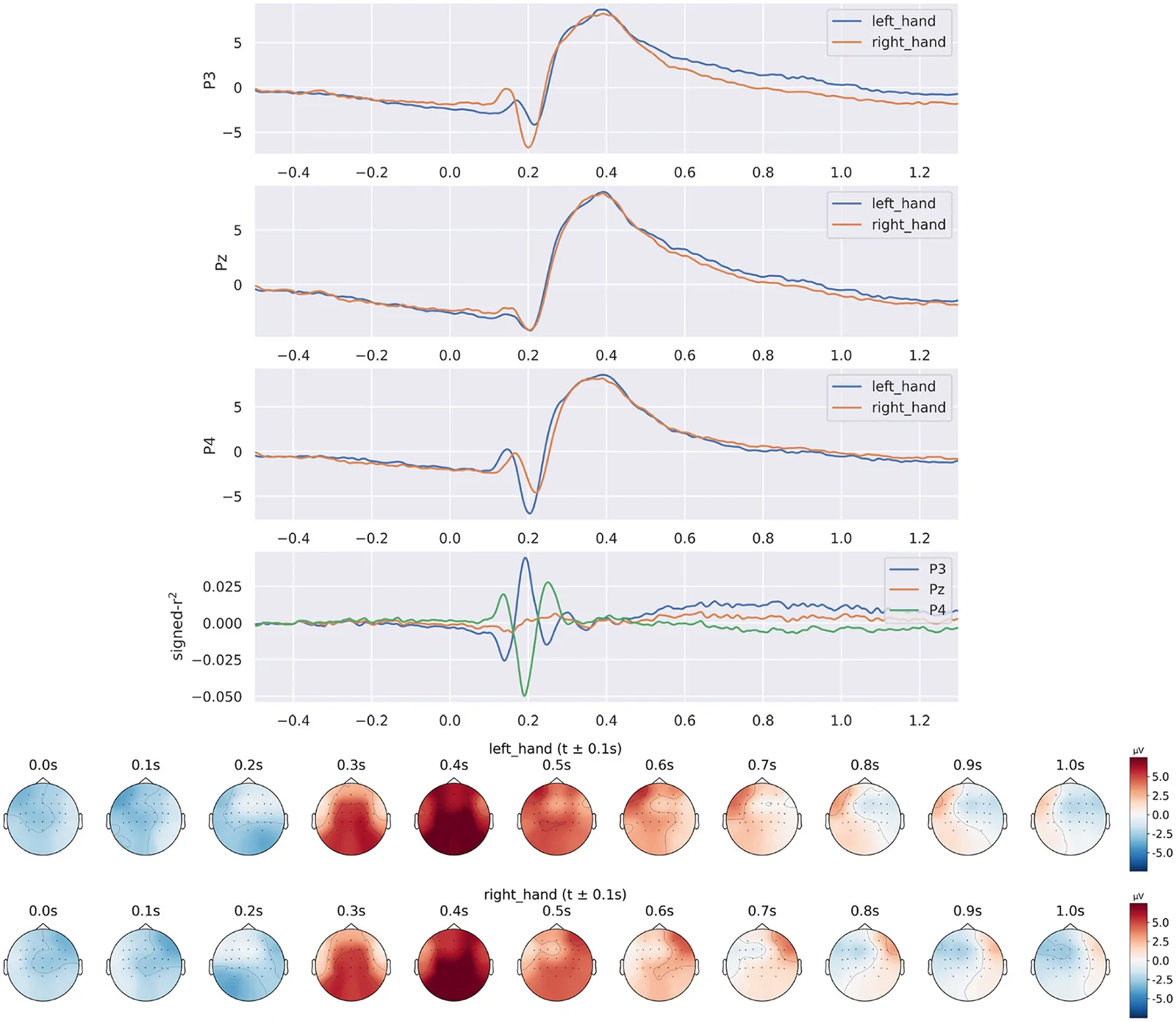
Grand-average ERP waveforms and signed-*r*^2^ values for the left- and right-hand classes in the Dreyer2023 dataset. From top to bottom, the ERP waveforms at channels *P*3, *P*_*z*_, and *P*4, followed by the signed-*r*^2^ values, are shown. The bottom row shows topographic maps of the ERP responses. For each time point, the topographic maps were computed by averaging the EEG signals over a time window of ±0.1 s.

**Figure 10:**
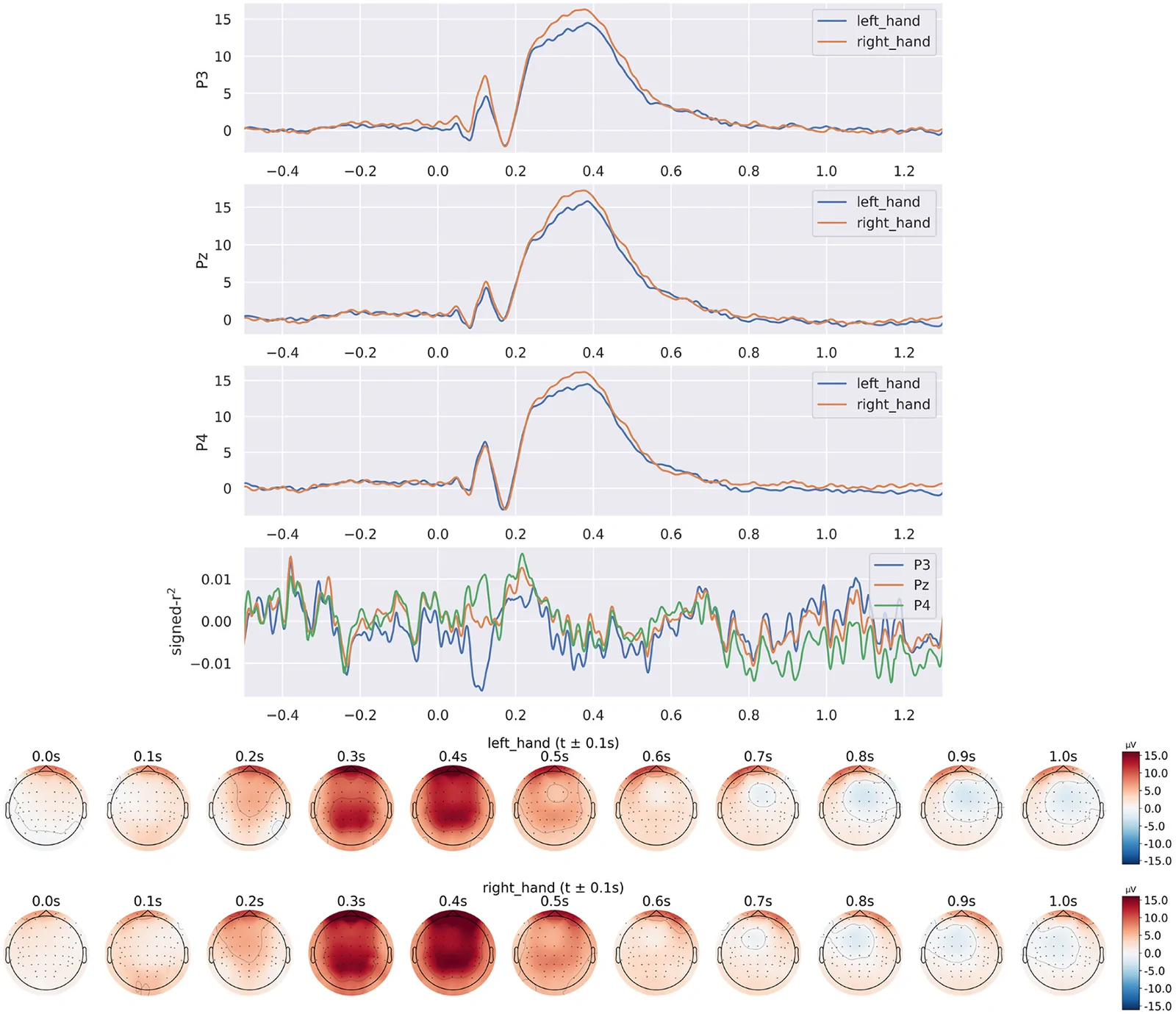
Grand-average ERP waveforms and signed-*r*^2^ values for the left- and right-hand classes in the Lee2019 dataset. From top to bottom, the ERP waveforms at channels *P*3, *P*_*z*_, and *P*4, followed by the signed-*r*^2^ values, are shown. The bottom row shows topographic maps of the ERP responses. For each time point, the topographic maps were computed by averaging the EEG signals over a time window of ±0.1 s.

For the Dreyer2023 dataset, contralateral VEPs were observed around 0.2 s, and the signed-*r*^2^ values indicated that this component exhibited high class separability.

For the Lee2019 dataset, no clear differences between classes were observed overall. However, a class-dependent difference was observed in the VEP at the P3 channel around 0.1 s, and the signed-*r*^2^ values suggested relatively high class separability at this latency. In contrast, the P300 component showed differences between the two classes around 0.2–0.5 s. However, the signed-*r*^2^ values remained relatively low during this time window, indicating that these differences did not correspond to high class separability.

Supplementary Figures S4, S5 present the ERP analysis results for the large- and small-difference groups, respectively. In both datasets, the differences in the VEP between classes (around 0.2 s for Dreyer2023 and 0.1 s for Lee2019) were more prominent in the large-difference group.

## 4 Discussion

These results demonstrate that including the visual cue onset in the epoching window affects classification accuracy differently depending on the model and approach used. In the cross-subject setting, the deep learning model performs better when the visual cue onset is included, whereas the CSP+LDA model performs worse under the same condition. In the within-subject setting, CSP+LDA shows no significant difference in mean classification accuracy between the two conditions. However, it is important to note the large inter-subject variability, some subjects achieving better performance with the visual cue onset, while others perform better without it. These inter-subject differences may have different causes. We can for instance hypothesize that some subjects pay more attention to the visual cues than others or that some subjects may have weaker ERD/ERS patterns during MI as compared to their visual-cue-induced brain responses (e.g., due to poorer MI abilities, or lower ERD/ERS signal-to-noise-ratio), leading classifiers to pick-up more easily the visual-cue-related brain patterns for these subjects, as compared to other subjects. The conducted ERP analyses seem to confirm these hypotheses. Indeed, users for which including the cue onset changed the most their classification accuracy (as compared to when not including this cue onset - i.e., the large-difference group) are the ones for which the cue onset-locked, early visual ERP is the most different between MI classes. This may suggest that these users were paying more attention to the cue, since attention is known to modulate visual ERPs (Hillyard and Anllo-Vento, 1998), and/or that the ERP signal-to-noise ratio of these users was the largest.

Nevertheless, these findings also highlight that classification accuracy alone is not a sufficient criterion for validating a model's effectiveness. Using attribution maps, we showed that although the deep learning model achieves higher accuracy when the visual cue onset is included in the epoching window, the features it relies on do not correspond to neurophysiological patterns of MI. Instead, we hypothesize, based on the analysis of the saliency maps, that the model predominantly learns to detect visual evoked potentials associated with the processing of the visual cue in the visual cortex. ERP analyses confirmed this hypothesis, with class-discriminant ERPs observed 0.1s or 0.2s after the visual cue onset, depending on the data set. This would lead to a useless MI-BCI classifier outside of this specific experimental setup. Indeed, in a real MI-BCI use scenario (whether synchronous or asynchronous), there is no visual cue instructing the subject what to do, since users decide by themselves what MI task to perform to control the system. A BCI classifier predominantly exploiting such cues would thus mostly fail in a real use case. In BCI scenarios where visual instructions cue may still be present, e.g., for MI-BCI-based stroke rehabilitation, having a classifiers exploiting attention to visual cues rather than motor-related patterns may also negatively impact the therapy, by rewarding stronger visual attention to cues rather than stronger ERD/ERS, the latter being the target for post-stroke motor rehabilitation based on BCI. Note however, that the brain response to these visual instructions cue is a real neurophysiological response (notably an ERP), and not an artifact. As such, including these visual instruction cues is a confounding factor in current MI-BCI applications, but the related brain responses may prove useful for other BCI applications, for which a reliance on cue-related activity does not disrupt the BCI objective.

Visualizing the CSP patterns in the within-subject setting confirms that including the visual cue onset has minimal influence on the extracted features, which aligns with the similar classification performance observed. However, we do not recommend including the visual cue since it leads to the emergence of CSP patterns localized in parietal areas for a few users. In contrast, in the cross-subject setting, the CSP patterns differ between the two conditions. In the condition without visual cue onset, two patterns (e.g., CSP2 and CSP4) weakly extract features from the sensorimotor cortex (C3 and C4), whereas in the condition with visual cue onset, patterns corresponding to MI-related activity are weaker and less clear. Despite the fact that cross subject training approach for CSP+LDA is rarely used in online setup, its evaluation allows to methodologically compare the influence of cross subject training and the choices of the model. It should be noted however, that the introspection methods used to investigate the features used by each model differ between models. Indeed, attribution maps for deep learning assess how perturbations of the input affect the deep learning outputs (and the maps we showed are averaged across trials) whereas CSP patterns reflect spatial projections of the discriminative CSP spatial filters, and as such are independent of the test input data, contrary to attribution maps. As such, comparing the resulting interpretations between CSP and deep learning should take into account these considerations: differences between them might also arise from different methods to identify the used features.

This difference in the nature of the learned features can be attributed to the model specificities. Deep learning models apply both spatial and temporal filtering, which makes them more adept at capturing transient visual evoked potentials, while CSP applies only spatial filtering and does not prioritize any specific time period, as power of the spatially filtered signal is averaged across all the epoching window. As a result, the deep learning model can focus more on the early visual processing phase (where ERPs are present) and less on the sustained motor imagery activity, while the CSP model distributes attention evenly across the entire trial duration, therefore extracting patterns present across all the epoch. Moreover, CSP is actually not able to extract ERP since it is based on the mean power of the signal, and not its time course (Lotte, 2014). Additionally, the preprocessing used differed between both methods (CSP+LDA and Deep Learning). Indeed, the input EEG was band-pass filtered in 8-30 Hz for the CSP+LDA, as recommanded for this algorithm which focused on sensorimotor rhythm Blankertz et al. (2008), whereas DeepConvNet input EEG was band-pass filtered in 0.5–40Hz, a broad band, which is also recommended for EEG Deep Learning, to let the network exploits as much information as possible, since deep learning can learn any representation Schirrmeister et al. (2017) (which CSP+LDA cannot do). This difference in preprocessing, while the appropriate one for each method—and the commonly used ones, can also explain differences in the algorithms sensitivity to visual-cue-onset-related bias. Indeed, we could show that visual-cue-onset led to specific, class-discriminant ERPs. ERPs are usually slow waves, i.e., components visible in low frequency bands. As such, they are most likely largely filtered out with CSP+LDA (using frequencies over 8Hz), but not with DeepConvNet, which uses low frequencies (≥ 0.5Hz) as well. This is likely one more reason why Deep Learning was more affected by confounding cue-related ERPs, contrary to CSP+LDA.

In this study we showed that the position of the epoching window can bias deep learning approach. Interestingly, among all the deep learning model hyperparameters, the size of the input window can also have a strong influence on classification accuracy. The more time points used as input, the higher the classification accuracy. This is confirmed with CSP when we compare the accuracy reported in this paper for 4-s window starting the epoching at 0.5 seconds (67.9%) and the one reported in the original dataset paper (Dreyer et al., 2023) with 1 seconds epochs and 15/16 s overlap from 0.5 s to 4.5 s (63.4%). This is the case even though the number of epochs which the model is trained with is much higher in the second condition (80 trials compared to 3,920). This observation is also the case with deep learning models, where a recent competition selected large input windows to perform best. In the NeurIPS 2021 BEETL Competition (Wei et al., 2022) the tutorial code proposed to epoch the signal directly at the beginning of the MI-trial, which may include visual indication cue. Even though the competition included multiple datasets together, which might help to reduce this bias, the results might be partially biased due to the visual cue. It is a good practice for a machine learning approach to choose the largest window possible, but they must not be located outside of the measured task, as this could lead to the measurement of other markers induced by the experimental environment, such as the visual cue at the start of the task, or the reward at the end. Also, the choice of a large epoching window differs from the design choice of most online BCI settings, where a fast and consistent feedback is important for allowing the user to perform better (Pfurtscheller and Neuper, 2001; Frolov et al., 2021). Therefore, in this context, small epoching windows are often used in order to give continuous feedback to the user. It should also be stressed that in our study, changing the input time window to exclude the visual cue onset was the only effective remedy to avoid the visual cue bias. Indeed, removing visual area channels (e.g., occipital or paretial EEG channels) from the deep network input did not prevent the visual cue onset bias, with still much higher accuracy when including the cue onset than when not doing so. This is likely due to volume conduction effects, with ERPs originating from visual areas of the brain spilling over non-visual-related EEG channels. Interestingly, our study used a fixed time window to avoid the visual cue bias (here, time windows starting 0.5s after the visual cue onset), based on the literature. ERP analyses also showed that this time window was practically appropriate to avoid the visual-cue-onset bias in those data sets. However, ERP analyses also suggested that the cue-induced class-discriminant ERPs may have different latencies depending on the data set [around 0.2 s for (Dreyer et al. 2023), around 0.1 s for (Lee et al. 2019)], probably due to different visual stimulus characteristics (e.g., different spatial locations for left and right cues in (Dreyer et al. 2023) but not in (Lee et al. 2019), a colored cue in (Dreyer et al. 2023) but a black-and-white one in (Lee et al. 2019), etc.). Thus, in order to best define the input time window to use for a given data set—to avoid the cue-related bias but use as much EEG information as possible - we would recommand conducting ERP analyses such as the ones conducted here. In particular, we would recommand estimating the cue onset-locked visual ERPs per MI class, and to estimate the discriminability of each time point in those ERPs (e.g., using signed-*r*^2^, as here). This would allow to select a time window excluding time points with class-discriminant visual ERPs.

These observations underscore the importance of evaluating not only classification performance but also the neurophysiological validity of the features extracted. When optimizing classification through methods such as grid search or Bayesian optimization, researchers should also assess the alignment of the learned representations with known brain dynamics. Improvements in accuracy that result from exploiting confounding signals—such as visual evoked potentials (ERPs) triggered by task-related cues—may lead to misleading conclusions and limit model generalization to real-world applications. Indeed, such visual cues are often present only in controlled laboratory experiments and are typically absent in real-world MI-BCI scenarios, where users initiate commands without external prompts and sometimes asynchronously. We thus stress that, in MI-BCI paradigms, Deep Learning models should NOT use input time windows that include the instruction visual cues onset time. Altogether, the effectiveness of machine learning/deep learning in BCI applications is therefore closely tied to the design of the experimental protocol, which should incorporate well-chosen paradigms, minimize confounding factors, and ensure that the models are trained on task-relevant brain activity.

We thus recommend that researchers systematically evaluate their BCI machine learning/deep learning models using XAI tools in order to validate their neurophysiological relevance and real world applicability. These evaluations should be included in submitted manuscripts, or at the very least made available to reviewers, to ensure that the proposed models are not merely exploiting dataset-specific biases.

This is particularly important for articles proposing new machine/deep learning methods, especially when these methods are benchmarked against state-of-the-art approaches using a shared preprocessing pipeline. Including XAI analysis in such cases is crucial to verify that no systematic bias is introduced by the preprocessing pipeline itself, and to ensure that the improved performance of the new model arises from its ability to extract neurophysiologically relevant features, rather than exploiting a confounding factor.

## 5 Conclusion

In this article, we investigated the impact of including the visual cue period in the epoching window on classification performance and feature extraction for two widely used approaches in MI-BCI: a classical pipeline using CSP+LDA, and deep learning-based models (DeepConvNet Schirrmeister et al., 2017 and REVE El Ouahidi et al., 2025). Our analysis revealed that the inclusion of the visual cue affects the two types of models differently.

For the deep learning models, including the visual cue led to a significant improvement in classification accuracy. However, attribution analysis demonstrated that the model's enhanced performance was largely driven by a confounding factor, i.e., the processing of the visual cue—in particular, class-discriminant visual evoked potentials related to this cue—rather than by motor imagery-related features such as ERD. This raises concerns about the neurophysiological validity of the model's decisions when trained under such conditions.

In contrast, the CSP+LDA model showed only minimal differences in performance between the two epoching conditions (with or without the visual cue onset) in the within-subject setting, with notable inter-subject variability. In the cross-subject setting, performance was slightly better when the visual cue onset was excluded. CSP pattern analysis further confirmed that including the visual cue onset sometimes led to the extraction of features unrelated to motor imagery, particularly in the parietal areas, which is inconsistent with the expected sensorimotor activity.

Overall, these findings highlight that improvements in classification accuracy should not be considered alone. The inclusion of visual cues can introduce confounding factors that may lead models, and especially deep learning architectures, to exploit irrelevant but discriminative signals. Such bias compromises the interpretability and generalizability of BCI systems, particularly in real-world or asynchronous use cases where visual cues are absent. A machine learning/deep learning model exploiting them is thus bound to fail. We thus urge BCI researchers using/designing EEG deep learning models to ensure their input EEG data does not include periods during which visual instruction cues are presented.

We emphasize the importance of incorporating explainability tools, such as attribution maps (e.g., Saliency maps) and CSP pattern analysis, into the evaluation pipeline of BCI models. These methods provide crucial insights into the neurophysiological relevance of the extracted features and help ensure that performance improvements are aligned with meaningful brain activity. Future research should continue to prioritize the interpretability and ecological validity of BCI systems, especially when leveraging increasingly complex models.

## Data Availability

Publicly available datasets were analyzed in this study. This data can be found here: https://www.nature.com/articles/s41597-023-02445-z; https://academic.oup.com/gigascience/article/8/5/giz002/5304369?login=false. The Python code to reproduce the results presented in this paper is available for free and open source in the following repository: https://github.com/dtrocellier/MI_bci_epoch_window_influence.
