# Supplementary material for: Visual cues in MI-BCI induce biases in EEG classification models with deep learning but not with standard machine learning
Source: Front Neuroergon. 2026 Sep 28;7:1804143. doi: 10.3389/fnrgo.2026.1804143 (PMC13656310; doi:10.3389/fnrgo.2026.1804143)
Supplement: Supplementary file 1 [file Supplementary_file_1.pdf]

# *Supplementary Material*

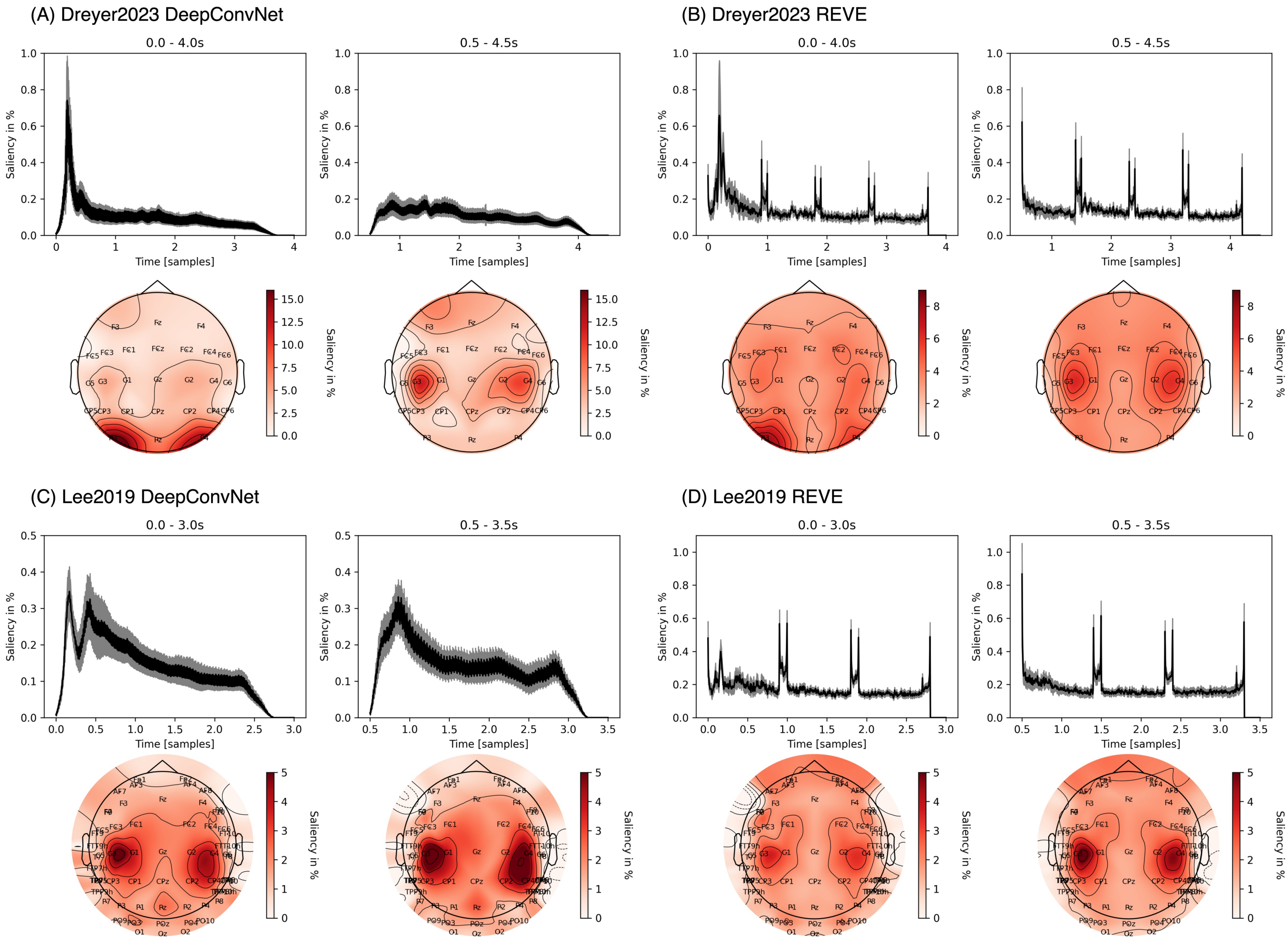


**Figure S1.** Temporal and spatial Integrated Gradients (IG) attribution maps for cross-subject training with DeepConvNet and REVE. (A) Dreyer2023 with DeepConvNet, (B) Dreyer2023 with REVE, (C) Lee2019 with DeepConvNet, and (D) Lee2019 with REVE. For each subfigure, the left panel shows the attribution maps obtained in the condition with the visual cue onset,, whereas the right panel shows the attribution maps obtained in the condition without the cue onset. For the temporal attribution maps, the black line indicates the mean attribution across subjects, and the gray shaded area indicates the standard deviation. For the spatial attribution maps, red regions indicate stronger attribution values.

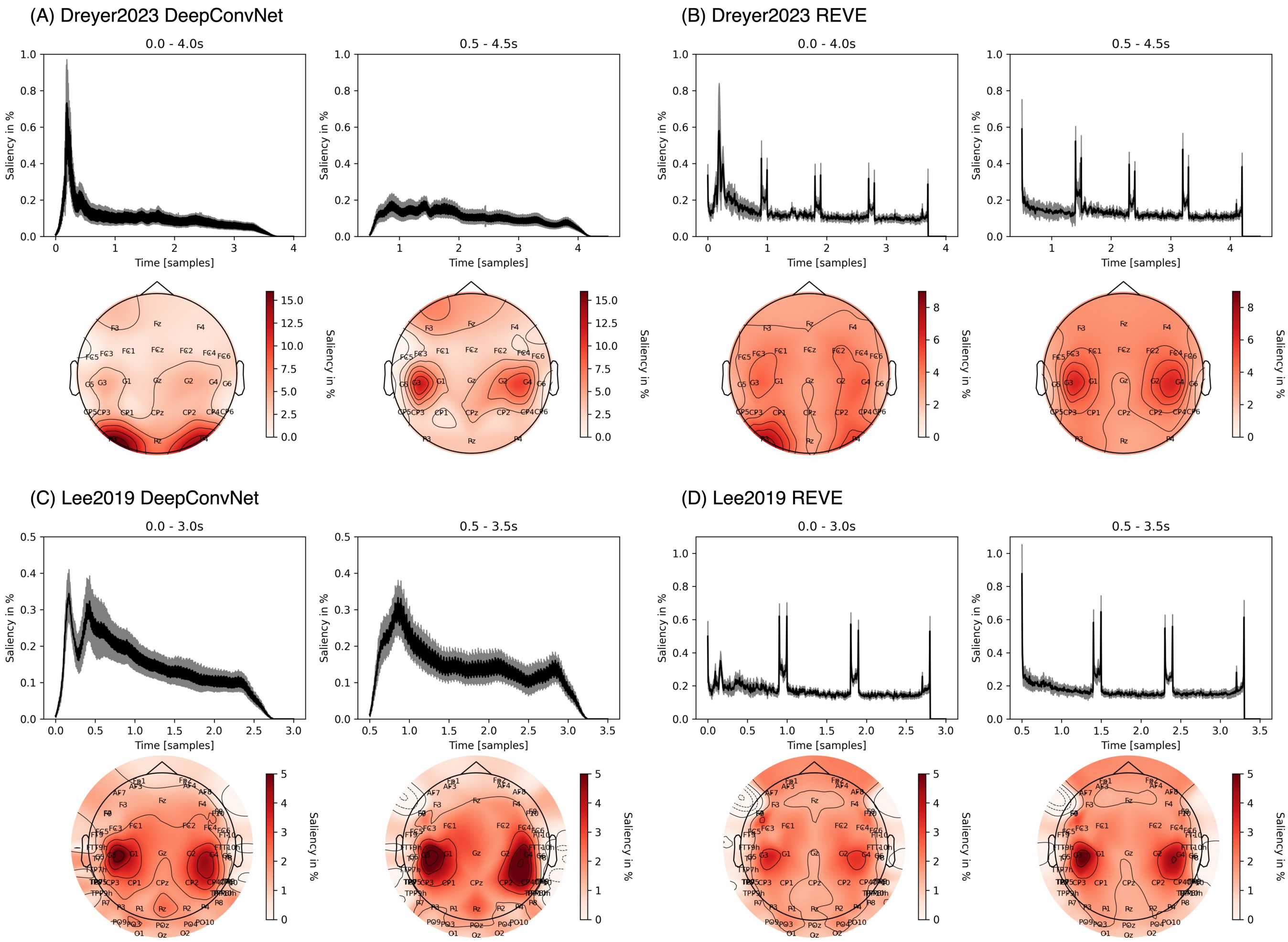


**Figure S2.** Temporal and spatial DeepLIFT attribution maps for cross-subject training with DeepConvNet and REVE. (A) Dreyer2023 with DeepConvNet, (B) Dreyer2023 with REVE, (C) Lee2019 with DeepConvNet, and (D) Lee2019 with REVE. For each subfigure, the left panel shows the attribution maps obtained in the condition with the visual cue onset, whereas the right panel shows the attribution maps obtained in the condition without the cue onset. For the temporal attribution maps, the black line indicates the mean attribution across subjects, and the gray shaded area indicates the standard deviation. For the spatial attribution maps, red regions indicate stronger attribution values.

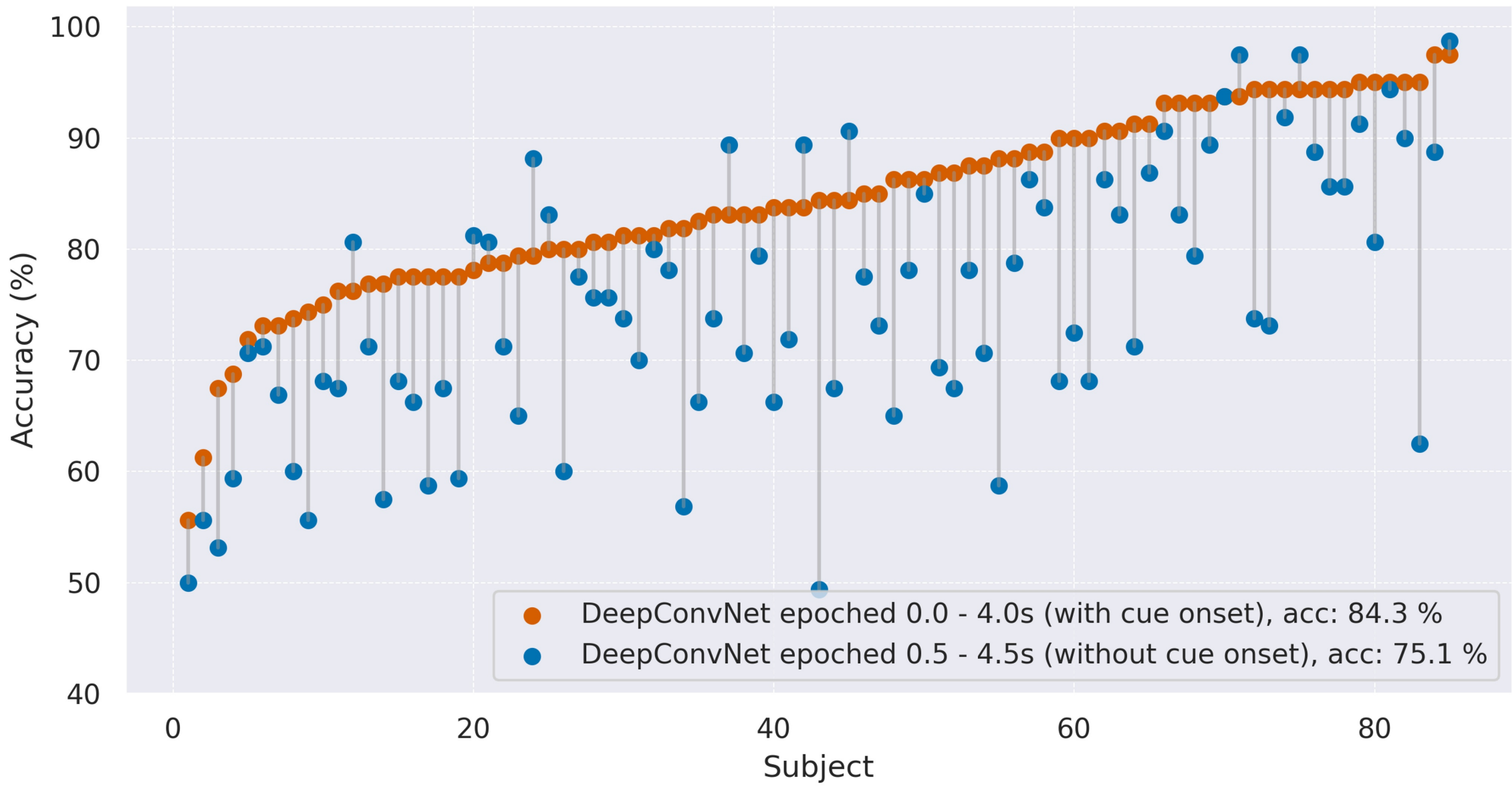


**Figure S3.** Accuracy of the DeepConvNet for the cross-subject condition in the Dreyer2023 dataset after removing the parietal channels ($P_3$, $P_z$, and $P_4$), shown for each subject using two different epoching approaches, with and without visual cue onset in the epoching window (orange and blue, respectively). Subjects are ordered by classification accuracy in the condition with visual cue onset.

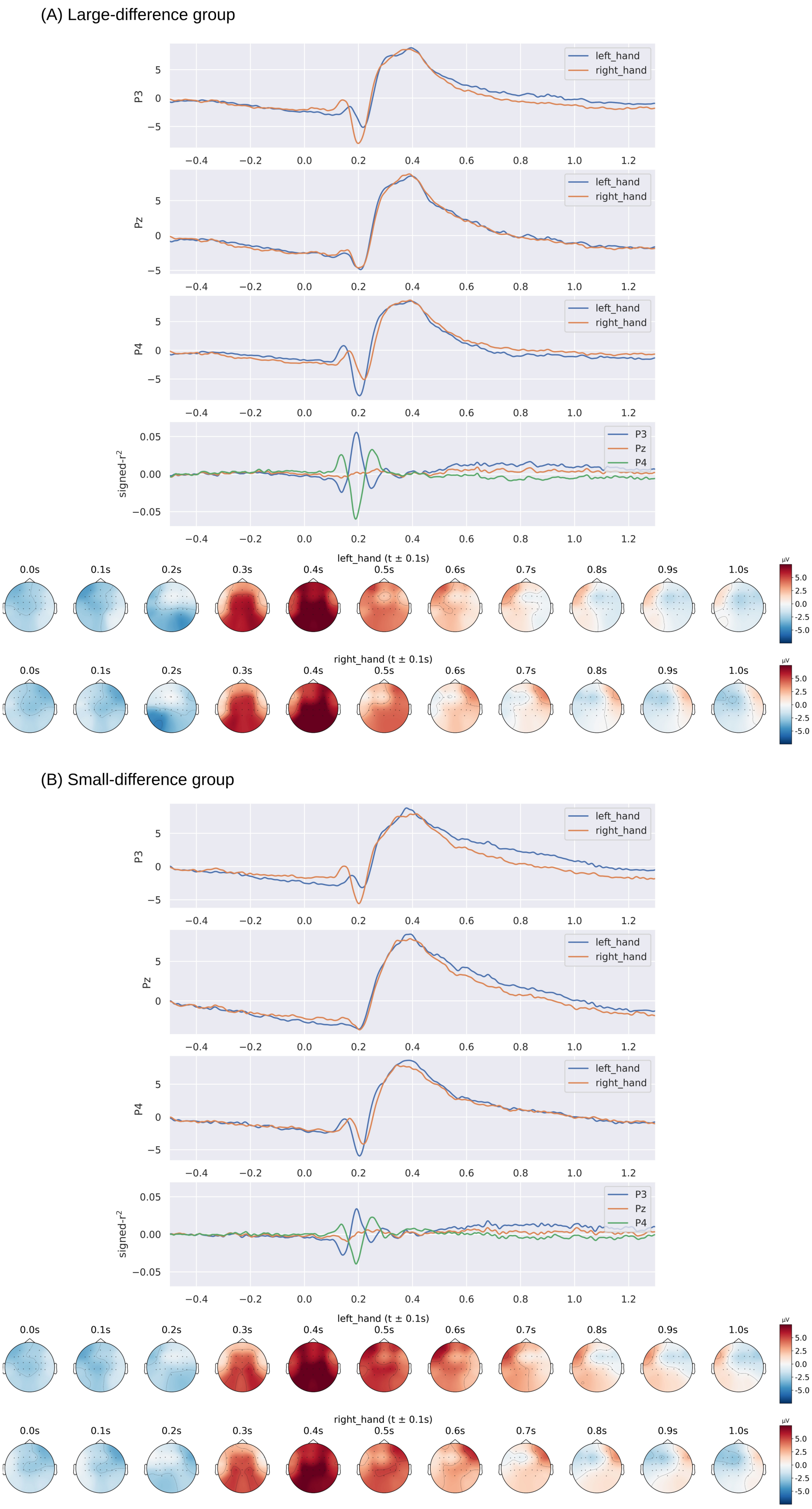


**Figure S4.** Grand-average ERP waveforms and signed-$r^2$ values for the left- and right-hand classes in the Dreyer2023 dataset. (A) Large-difference group and (B) Small-difference group. For each panel, from top to bottom, the ERP waveforms at channels $P3$, $P_z$, and $P4$, followed by the signed-$r^2$ values, are shown. The bottom row shows topographic maps of the ERP responses. For each time point, the topographic maps were computed by averaging the EEG signals over a time window of $\pm 0.1$ s.

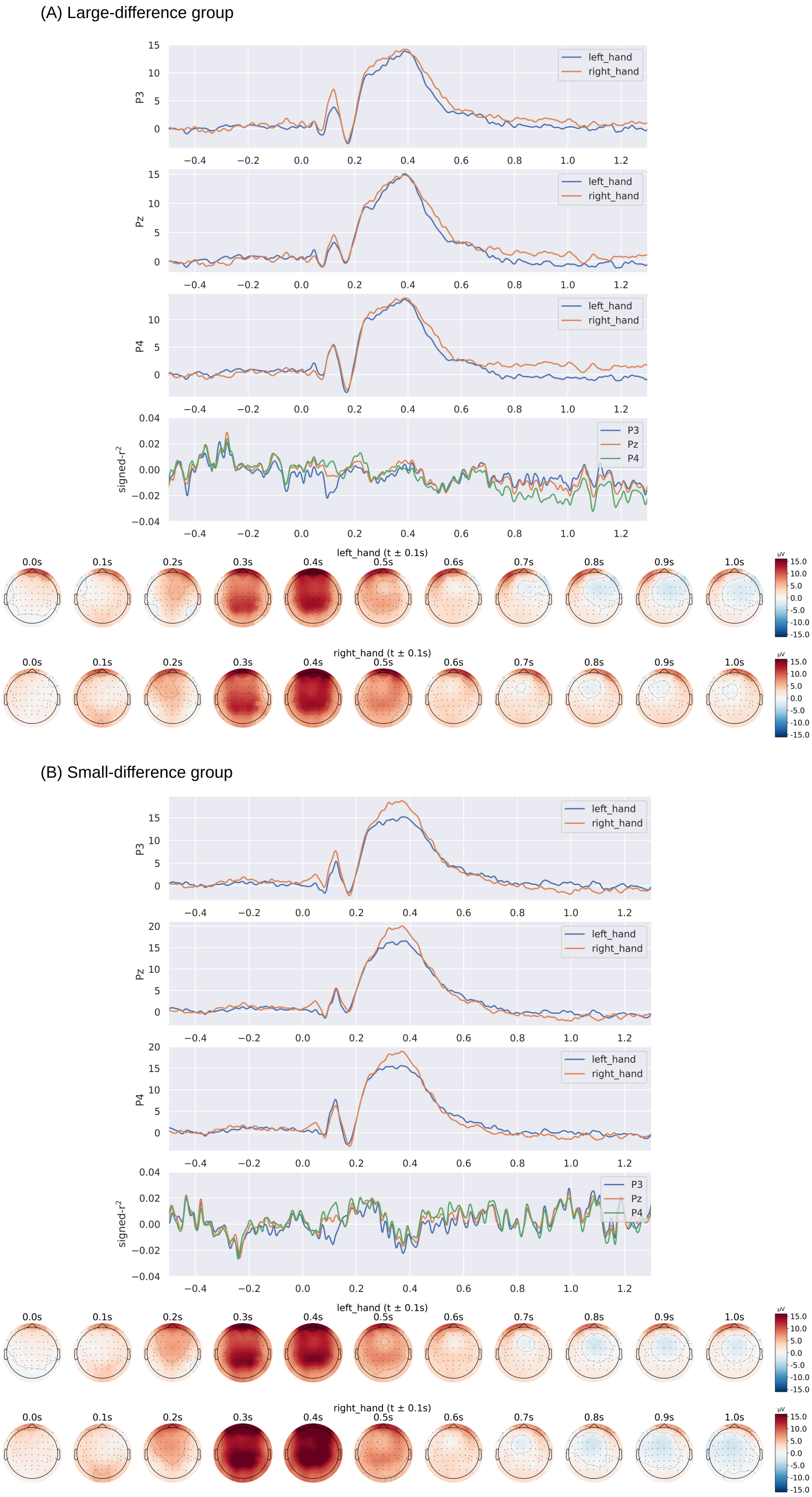


**Figure S5.** Grand-average ERP waveforms and signed-$r^2$ values for the left- and right-hand classes in the Lee2019 dataset. (A) Large-difference group and (B) Small-difference group. For each panel, from top to bottom, the ERP waveforms at channels $P3$, $P_z$, and $P4$, followed by the signed-$r^2$ values, are shown. The bottom row shows topographic maps of the ERP responses. For each time point, the topographic maps were computed by averaging the EEG signals over a time window of $\pm 0.1$ s.
